# Supplementary material for: Targeting Aberrant Epigenetic Networks Mediated by PRMT1 and KDM4C in Acute Myeloid Leukemia
Source: Cancer Cell. 2016 Jan 11;29(1):32–48. doi: 10.1016/j.ccell.2015.12.007 (PMC4712026; doi:10.1016/j.ccell.2015.12.007)
Supplement: Document S1. Supplemental Experimental Procedures, Figures S1–S6, and Tables S1–S3 [file mmc1.pdf]

Cancer Cell, Volume *29*

Supplemental Information

Targeting Aberrant Epigenetic Networks Mediated by PRMT1 and KDM4C in Acute Myeloid Leukemia

Ngai Cheung, Tsz Kan Fung, Bernd B. Zeisig, Katie Holmes, Jayant K. Rane, Kerri A. Mowen, Michael G. Finn, Boris Lenhard, Li Chong Chan, and Chi Wai Eric So

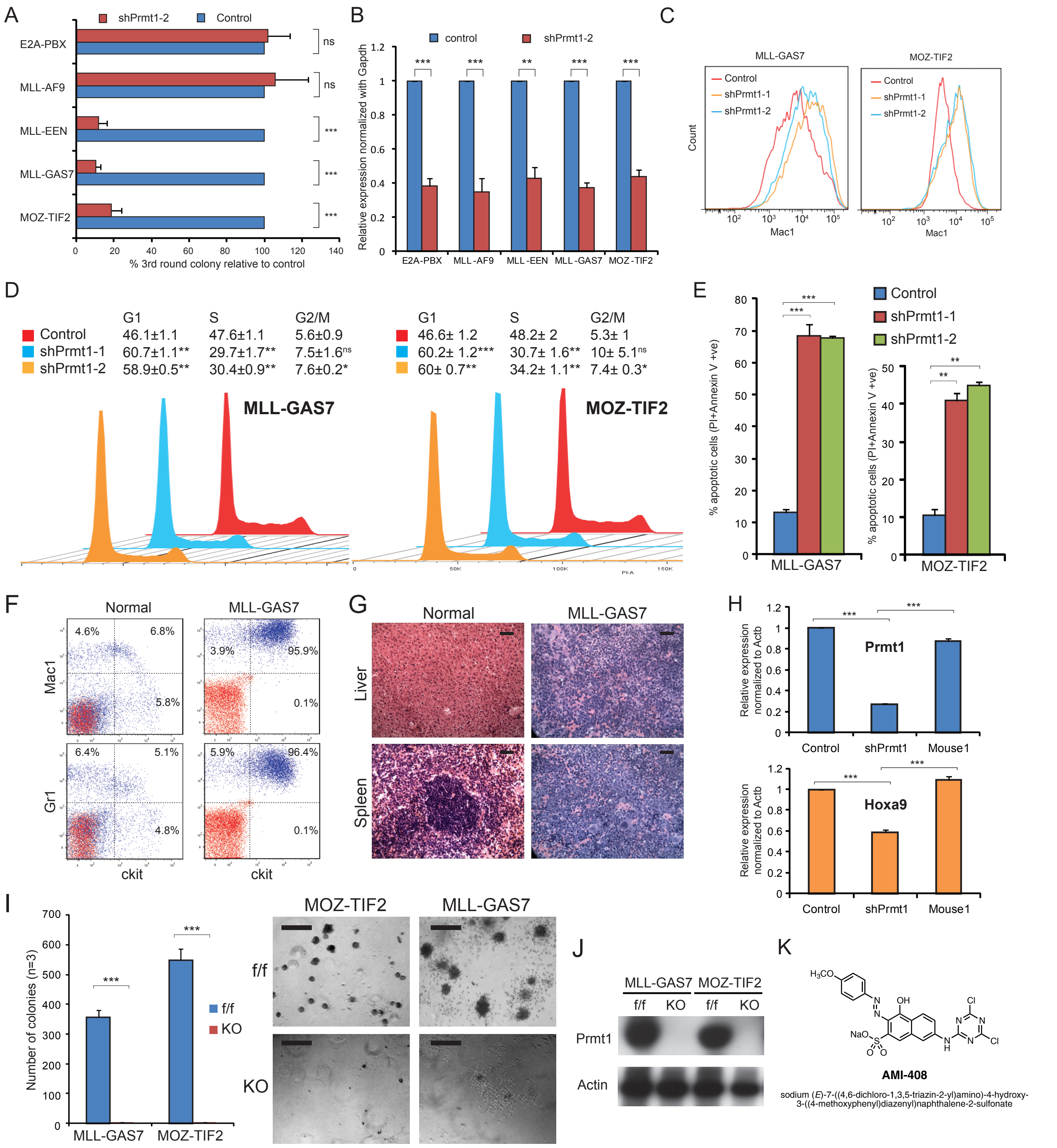

Figure S1, related to Figure 1. Effect of Prmt1 knockdown and AMI-408 treatment on leukemogenesis

A. Prmt1 knockdown using shPrmt1-2 specifically suppresses MLL-EEN, MLL-GAS7 and MOZ-TIF2 colony formation in replating assay. Shown are mean and SD (n=3).

B. RT-qPCR validation of Prmt1 knockdown by shPrmt1-2 in leukemic cells as indicated. Shown are mean and SD (n=3).

C. Prmt1 knockdown in MLL-GAS7 and MOZ-TIF2 leukemia cells induces the up-regulation of myeloid surface marker Mac1 expression as revealed by FACS analysis.

D. Cell cycle analysis revealed an increased in G1 and decreased S phase population in both MLL-GAS7 and MOZ-TIF2 leukemia cells after Prmt1 knockdown. Shown are mean and SD (n=3).

E. Prmt1 knockdown in MLL-GAS7 and MOZ-TIF2 leukemia cells with two independent Prmt1 shRNA results in increased apoptotic cell death as revealed by Annexin V-PI staining. Shown are mean and SD (n=3).

F. FACS analysis of the bone marrow of normal, MLL-GAS7 leukemic mice showing the expression of c-kit and both myeloid markers Mac1 and Gr1 in leukemic blasts.

G. Histological analysis of H&E tissue sections showing the infiltration of leukemic blasts into liver and spleen of leukemic mice as indicated. The normal spleen and liver are also shown as references. Scale bar indicate 25 μm.

H. Some of the mice transplanted with Prmt1 knockdown leukemia cells eventually developed acute myeloid leukemia with a longer latency and lower disease penetrance. Quantitative RT-PCR analysis in those leukemia cells (Mouse 1) revealed a comparable expression level of *Prmt1* and *Hoxa9* mRNA to control that indicative of the loss of Prmt1 down-regulation in those leukemia cells.

I. Prmt1 knockout (KO) in MLL-GAS7 and MOZ-TIF2 suppress colony formation in methylcellulose replating assay. “f/f” was served as control. Shown are mean and SD (n=3). Scale bar indicate 5 mm.

J. Prmt1 KO in both MLL-GAS7 and MOZ-TIF2 leukemia cells was confirmed by Western blot with anti-Prmt1 and actin antibodies.

K. Structure of compound AMI-408.

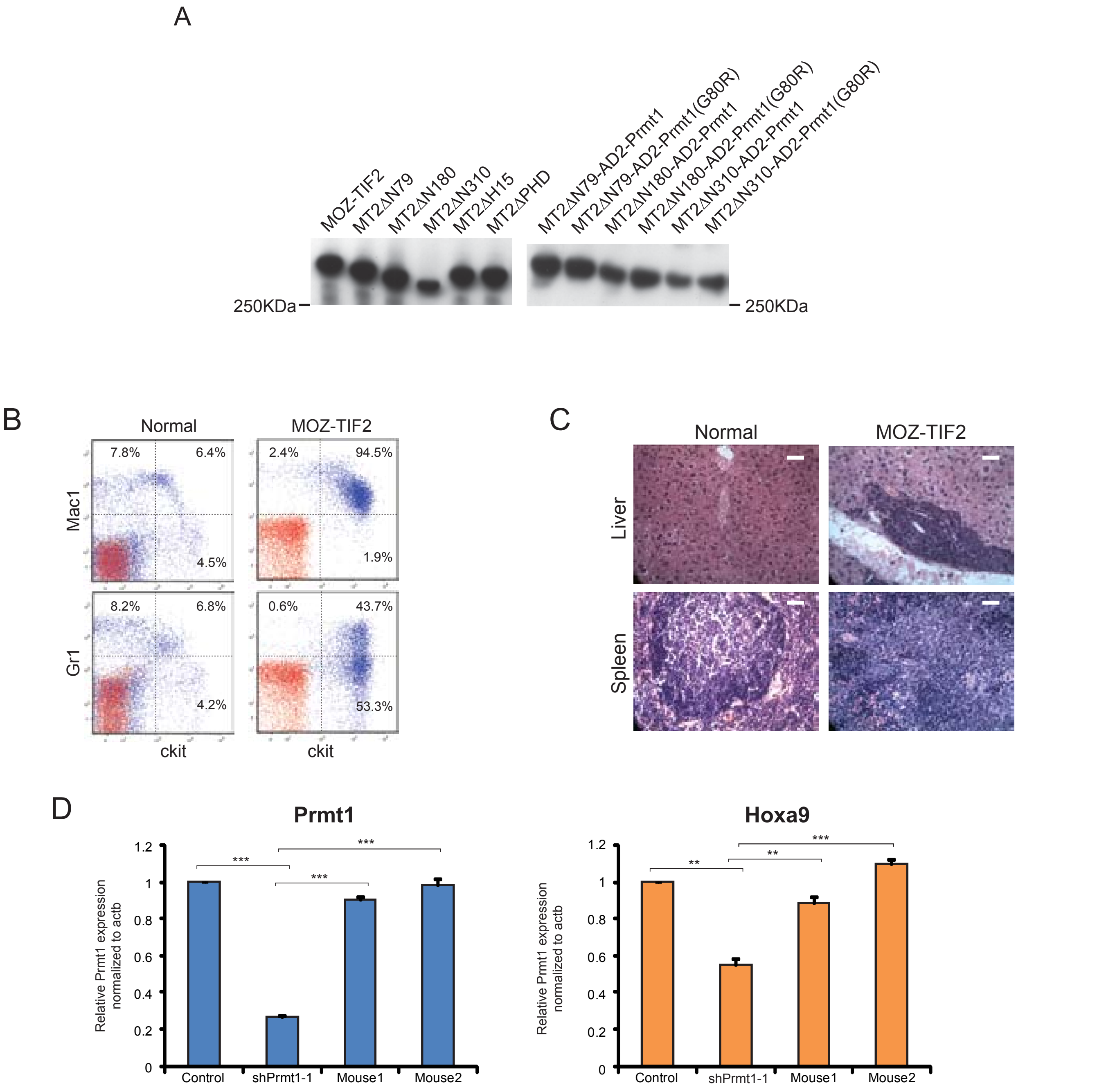

Figure S2, related to figure 2. Effect of Prmt1 knockdown on MOZ-TIF2 leukemia cells in vitro & in vivo

A. Western blot showed the expression of FLAG-tagged MOZ-TIF2, deletion mutants and its Prmt1 rescue fusions in transduced cells.

B. FACS analysis of the bone marrow of normal and MOZ-TIF2 leukemia mice showing the expression of c-kit and both myeloid markers Mac1 and Gr1 in leukemic blasts.

C. Examination of tissue sections show the infiltration of leukemic blasts into liver and spleen of leukemic mice. Scale bar indicate 25 μM.

D. Some of the mice transplanted with Prmt1 knockdown leukemia cells developed acute myeloid leukemia with a longer latency and lower disease penetrance. Quantitative RT-PCR analysis of those leukemia cells (Mouse 1 and 2) revealed a comparable expression level of *Prmt1* (left panel) and *Hoxa9* (right panel) mRNA to control that indicative of the loss of Prmt1 down-regulation in those leukemia cells.

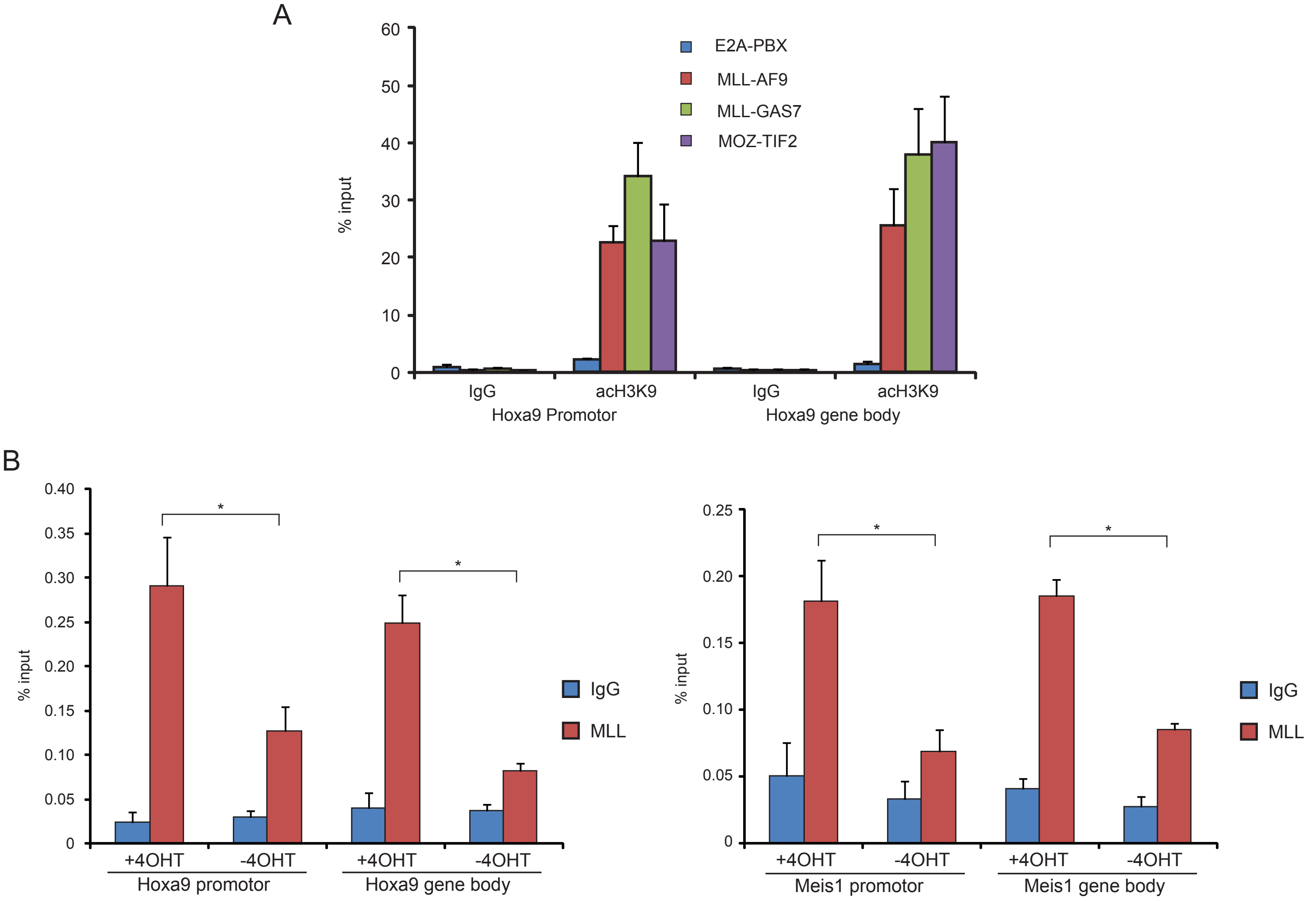

Figure S3, related to Figure 3. ChIP analysis of H3K9ac on leukemic cells and MLL binding at Hoxa9 loci in MLL-AF9-ER cells

A. Increased H3K9 acetylation in *Hoxa9* loci were detected in MLL-AF9, MLL-GAS7 and MOZ-TIF2 leukemic cells but not in E2A-PBX, as revealed by ChIP. Shown are mean and SD (n=3).

B. Significant reduction of MLL binding to *Hoxa9* and *Meis1* loci was detected in MLL-AF9-ER transformed cells 4 days after tamoxifen (4OHT) withdrawal, as revealed by ChIP using anti-MLL antibody. Shown are mean and SD (n=3).

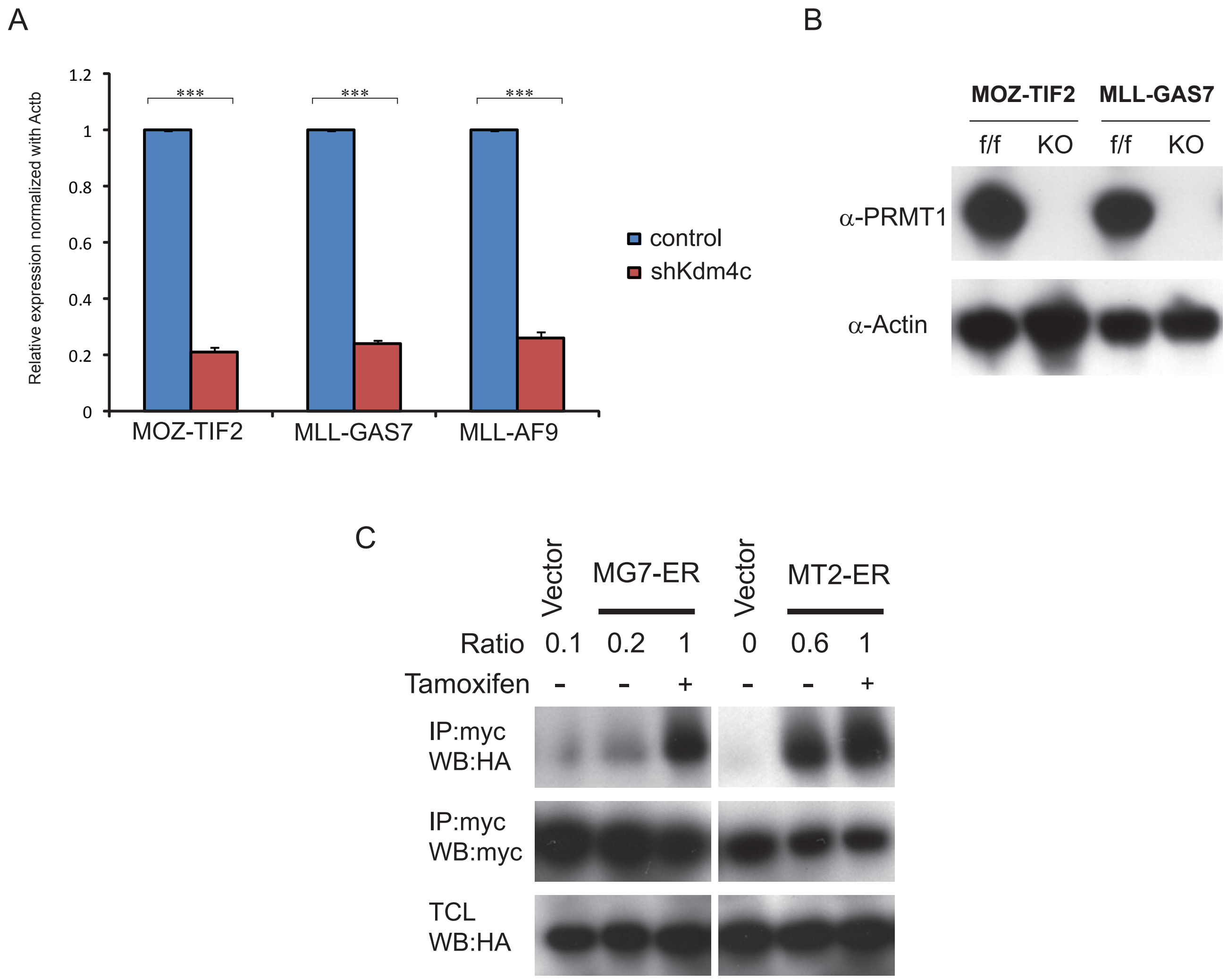

Figure S4, related to Figure 4. Validation of Kdm4c knockdown and Prmt1 knock-out in RNA-seq samples

A. RT-qPCR validation of Kdm4c knockdown (shKdm4c) on MOZ-TIF2, MLL-GAS7 and MLL-AF9 leukemic cells prior to RNA-Seq analysis. Shown are mean and SD (n=3).

B. Western blot shows the loss of Prmt1 protein expression in MOZ-TIF2 and MLL-GAS7 leukemic cells after knockout (KO), compared with floxed control (f/f).

C. FLAG tagged inducible MLL-GAS7-ER or MOZ-TIF2-ER were co-transfected with myc-KDM4C and HA-Prmt1 in HEK293 cells followed by co-immunoprecipitation analysis. Activation of MLL-GAS7 and MOZ-TIF2 leukemic fusions by tamoxifen resulted in increasing co-recruitment of Prmt1 by Kdm4c comparing with inactive complexes. The band intensity was normalized to control cells expressing active leukemic fusions.

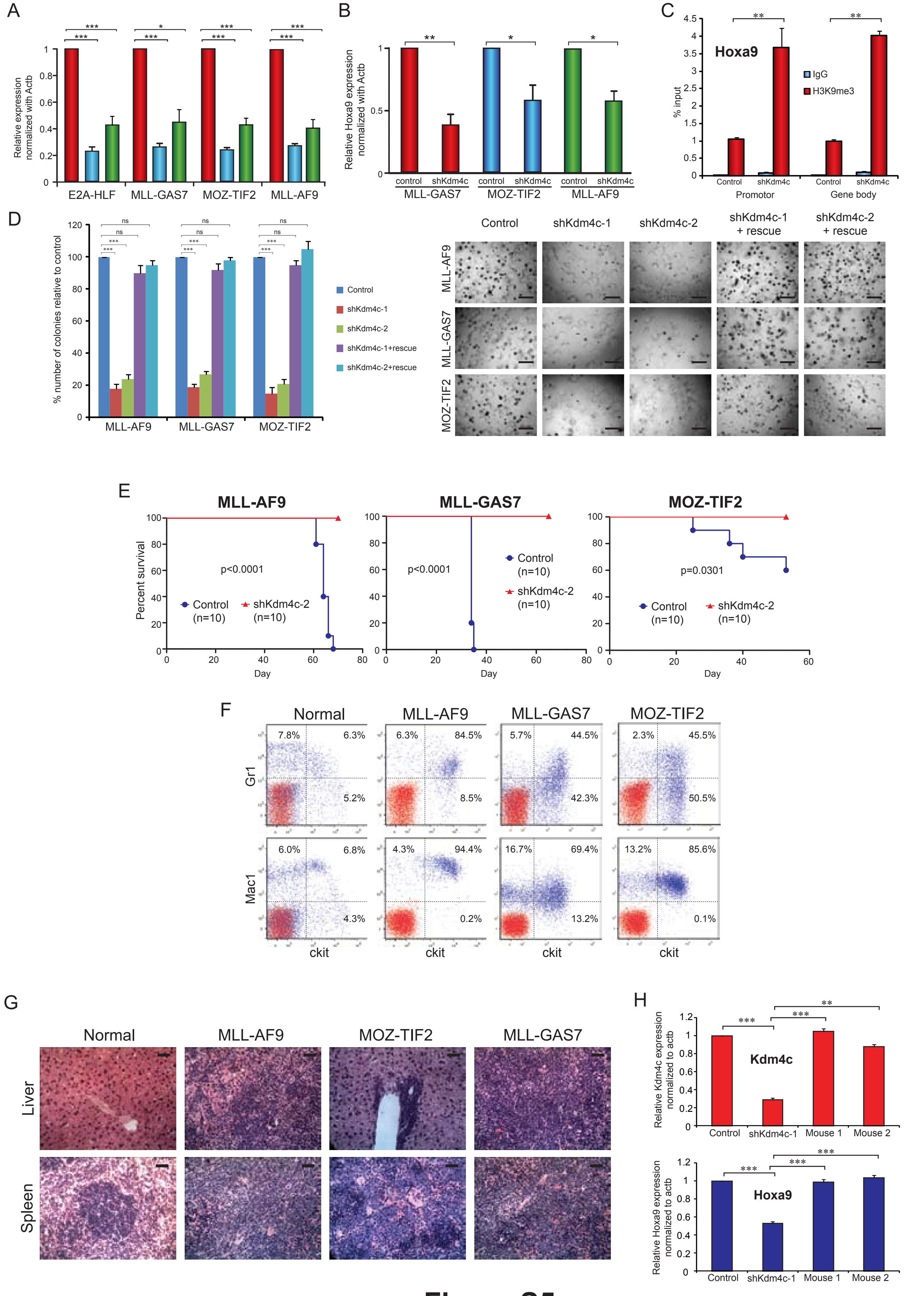
A
Relative expression normalized with Actb
E2A-HLF
MLL-GAS7
MOZ-TIF2
MLL-AF9
B
Relative Hoxa9 expression normalized with Actb
control
shKdm4c
MLL-GAS7
MOZ-TIF2
MLL-AF9
C
Hoxa9
IgG
H3K9me3
% input
Control
shKdm4c
Promotor
Gene body
D
% number of colonies relative to control
Control
shKdm4c-1
shKdm4c-2
shKdm4c-1+rescue
shKdm4c-2+rescue
MLL-AF9
MLL-GAS7
MOZ-TIF2
Control
shKdm4c-1
shKdm4c-2
shKdm4c-1 + rescue
shKdm4c-2 + rescue
E
MLL-AF9
MLL-GAS7
MOZ-TIF2
Percent survival
p<0.0001
p<0.0001
p=0.0301
Control (n=10)
shKdm4c-2 (n=10)
Day
F
Normal
MLL-AF9
MLL-GAS7
MOZ-TIF2
Gr1
Mac1
ckit
G
Normal
MLL-AF9
MOZ-TIF2
MLL-GAS7
Liver
Spleen
H
Relative Kdm4c expression normalized to actb
Kdm4c
Relative Hoxa9 expression normalized to actb
Hoxa9
Control
shKdm4c-1
Mouse 1
Mouse 2

Figure.S5

Figure S5, related to Figure 5. Effect of Kdm4c knockdown on leukemic transformation in vitro and leukemogenesis in vivo

A. Quantitative RT-PCR analysis showing the efficient knockdown of *Kdm4c* by two different shKdm4c shRNAs in different leukemia cells. Shown are mean and SD (n=3).

B. Quantitative RT-PCR analysis showing the significant reduction of *Hoxa9* expression in MLL-GAS7, MOZ-TIF2 and MLL-AF9 after Kdm4c knockdown by shKdm4c-1.

C. ChIP analysis showing the increased H3K9me3 mark at *Hoxa9* loci upon Kdm4c knock-down in MLL-AF9 leukemic cells.

D. Suppression of colony formation induced by MLL-AF9, MLL-GAS7 and MOZ-TIF2 is rescued by the co-expression of shRNA resistant human KDM4C. Shown are mean and SD (n=3). Scale bars indicate 50 μm.

E. Knockdown of Kdm4c in leukemic cells by shKdm4c#2 resulted in a significant increase in disease latency in MLL-AF9 (log-rank test p<0.0001), MLL-GAS7 (p<0.0001), MOZ-TIF2 (p=0.0301) mediated leukemia. Median disease latency: MLL-AF9: 64 days; MLL-GAS7: 34 days; MOZ-TIF2: 56 days; shKdm4c: undefined.

F. FACS analysis of the bone marrow of normal and leukemic mice as indicated showed the expression of c-kit and myeloid surface markers Gr1 and Mac1 in leukemia blasts.

G. Histological analysis revealed the infiltration of leukemic blasts into the liver and spleen of leukemic mice as indicated. Scale bar indicate 25 μm.

H. Quantitative RT-PCR validation of Kdm4c knockdown in leukemia cells isolated from disease mice. Analysis of the expression level of *Kdm4c* mRNA revealed the escape of Kdm4c knockdown (upper panel) and the upregulation of *Hoxa9* expression (lower panel) in the MLL-AF9 leukemic cells (Mouse 1 and 2).

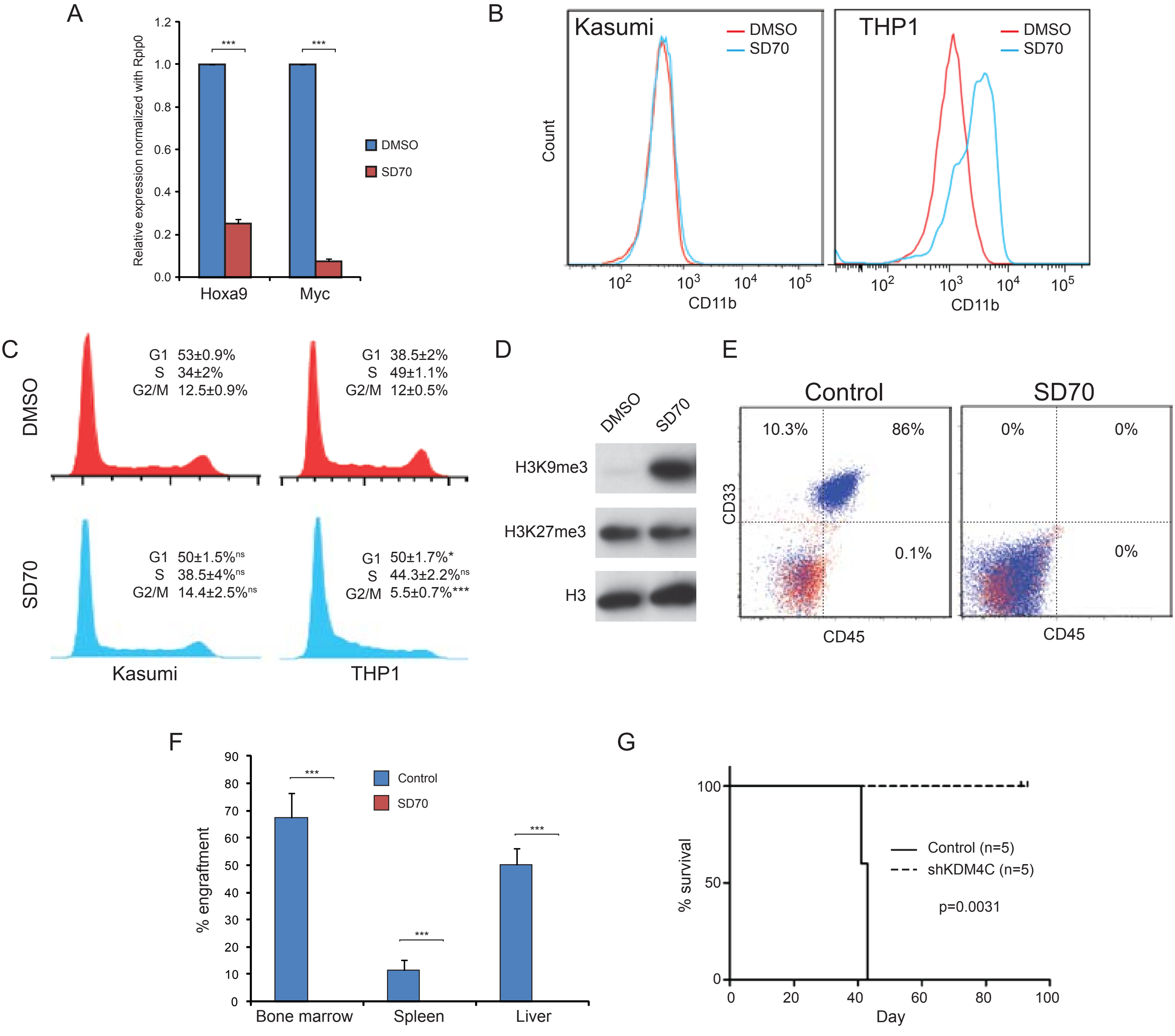

Figure S6, related to Figure 6. SD70 inhibits murine and human leukemogenesis.
A. Quantitative RT-PCR analysis showed that SD70 repressed the transcription activation of *Hoxa9* and *Myc* in murine MLL-AF9 leukemic cells after 3 days treatment. Shown are mean and SD (n=3).
B. Expression of myeloid surface marker CD11b was induced by SD70 after 3 days treatment in THP1 leukemia cells but not the control Kasumi cell lines, as revealed by FACS analysis.
C. Cell cycle analysis showing SD70 treatment as (B) induces an increase of G1 and reduction of S populations in THP1 carrying MLL-AF9 but not non-MLL rearranged Kasumi cells with AML1-ETO fusion.
D. Western blot of H3K9 and H3K27 methylation in SD70 treated human MLL leukemia cells for 3 days revealed the upregulation of H3K9me3 marks upon SD70 treatment as indicated in Figure 6H, which is consistent with the inhibition of KDM4C demethylation activity.
E. Representative FACS plot of bone marrow engraftment of human primary leukemia MLL3 in NSG mice from control and SD70 treated groups. They were analysed by FACS with human CD33 and CD45 staining. Engraftment of MLL3 leukemia cells was indicated by CD45+CD33+ population.
F. A summary of the percentage of MLL3 human primary leukemia engraftment in the bone marrow, spleen and liver in both the control and SD70 treatment cohort (n=5). SD70 treatment significantly inhibited the engraftment of human leukemia cells in the organs analysed (unpaired t-test; ***p<0.001).
G. Survival curves of in vivo xenograft study using MLL3 human primary leukemia cells transduced with scramble control or shKDM4C. NSG mice transplanted with MLL3 cells with scramble shRNA developed leukemia within 50 days, whereas cohort with KDM4C knockdown (shKDM4c) MLL3 cells had not succumbed to leukemia (log-rank test p=0.0031). Median disease latency: Control – 43 days; shKDM4C – undefined.

Supplemental Experimental Procedures

Plasmid constructs

Leukemia fusions and their deletion mutant constructs were cloned into MSCV retroviral vectors (Clontech) or pcDNA3-FLAG expression vector to generate FLAG-tagged expression plasmids. To generate myc-tagged expression constructs, genes of interest were subcloned into pCS2+MT vector. Most of the histone demethylase family genes were derived from IMAGE clones and subsequently subcloned into pRRL-3xFLAG lentiviral vector. Kdm4 demethylase family plasmids were kindly provided by Kristian Helin & Thomas Jenuwein; KDM3A/B were gifts of Zhang Yi. MSCV-MOZ-TIF2 is a kind gift from Brian Huntly. All plasmid constructs were confirmed by DNA sequencing. Prmt1 shRNA constructs were prepared as described (Cheung et al., 2007). Kdm4c shRNAs in pLKO lentiviral vector were kind gifts from Bill Hahn (shKdm4c TRCN0000103550, shKdm4c#2 TRCN0000103553 from Sigma Aldrich). Lentiviral vector harbouring luciferase reporter and hygromycin resistance gene (pCDH-CMV-luc-EF1-Hygro) was kindly provided by Lou Chesler.

Cell lines

Murine leukemia cell lines (E2A-PBX, MN1, MOZ-TIF2, MLL-AF9, MLL-ENL and MLL-GAS7) were established from retroviral transduction transformation assay (RTTA) as described (Zeisig and So, 2009) and cultured in RPMI with 20% FCS, 20% WEHI conditioned medium, 100 unit/ml penicillin and 100 μg/ml streptomycin (Sigma). Human leukemia cell lines NB4 was kindly provided by Arthur Zelent, THP1 by Mel Greaves, KASUMI by Olaf Heidenreich and K562, KG1, SEM, and HB11;19 were collected from ATCC. They were cultured in RPMI supplemented with 10% FCS, 100 unit/ml penicillin and 100 μg/ml streptomycin. Primary human patient samples (MLL1-3 and non-MLL1-3) were cultured in IMEM supplement with 10% FBS, 1X Glutamax (Invitrogen), 10ng/ml interleukin 3 (IL3), 10ng/ml interleukin 6 (IL6), 10ng/ml stem cell factor (SCF), 10ng/ml thrombopoietin (TPO), 10ng/ml FLT3 ligand (all human; Peprotech), 100 unit/ml penicillin and 100 μg/ml streptomycin.

Viral transduction and methylcellulose replating assay

RTTA was performed as previously described (Zeisig and So, 2009). Briefly, retroviral or lentiviral supernatants were collected 3 days after transfection of HEK293-GP cells (Clontech) or HEK293T respectively and used to transduce hematopoietic progenitors and stem cells positively selected for c-Kit expression by magnetic activated cell sorting (MACS; Miltenyi Biotec) from the bone marrow of 4-10 week old C57BL/6 mice or UbC-Luciferase transgenic mice kindly provided by Andrew Kung (Becker et al., 2006). After spinoculation by centrifugation at 500 $\times$ g for 2 hours at 32°C, transduced cells were cultured overnight in RPMI supplemented with 10% FCS, 20 ng/ml SCF, 10 ng/ml each of IL-3 and IL-6 (all murine; Peprotech). They were then plated in 1% methylcellulose (Stem Cell Technologies, Vancouver, BC, Canada) supplemented with the same cytokines plus 10 ng/ml GM-CSF (Peprotech) and 1 mg/ml geneticine or 30 μg/ml blasticidine (Life Technology) for positive selection of transduced cells. For co-transduction experiments of leukemia fusions with shRNA constructs, the cells were co-selected with 2 μg/ml puromycin and 1mg/mL geneticine. For Kdm4c knockdown rescue experiments, leukemia cells were cotransduced with Kdm4c shRNA and shRNA-resistant human KDM4C lentiviruses, and co-selected with 2 μg/ml puromycin and 30 μg/ml blasticidin. After 5-7 days culture colonies were counted to calculate the transduction efficiency. Single-cell suspensions (10^4 cells) of antibiotic resistant colonies were then replated in methylcellulose media supplemented with the same cytokines aforementioned without antibiotics. Subsequent replatings were usually repeated every 5-7 days.

In vivo Leukemogenesis assays

To generate full-blown murine leukemia cells ("leukemia cells" throughout the manuscript), 10^6 immortalized cells after the third round replating were injected via the tail vein into 6-10 weeks old syngeneic C57BL/6 mice which had received a sub-lethal dose of 5.25 Gy total body γ irradiation (^{137}Cs). To study the effect of down-regulation of Prmt1 and Kdm4c on leukemogenesis in vivo, the mice were injected with 10^5 murine full-blown leukemia cells as indicated. In vivo experiments with Prmt1 knockdown were transduced with either control vector or shPrmt1 retrovirus and sorted by eEGFP signal for transduced cells. Transduced Kdm4c knockdown cells (also control vector transduced) were antibiotic selected prior to transplantation. Prmt1 knockout achieved by tamoxifen

treatment were confirmed by PCR genotyping prior to transplantation. Mice were maintained and monitored for development of leukemia by FACS analysis. Tissues were fixed in buffered formalin, sectioned and stained with hematoxylin and eosin for histological analysis. For primary human sample MLL3, cells were transduced with control plasmid or shKDM4c plasmid. Transduced cells were antibiotic selected for 3 days and 10^5 selected cells were transplanted by intra-femoral injection into sublethally irradiated immunodeficient *NOD/SCID/IL2Rg-/-* (NSG) mice. For in vivo experiments involved bioluminescence imaging and quantification of the leukemia burden, murine leukemias were transformed in c-Kit positive bone marrow cells from Ubc-luciferase reportor C56BL6 mice (Becker et al., 2006). Human leukemia was transduced with a lenitiviral luciferase reporter as indicated in the plasmid constructs section. Transplanted mice were injected with 150 mg/kg D-luciferin substrate intraperitoneally and bioluminescence image acquired using IVIS Lumina II® (Caliper; Perkin Elmer) with software Living Image® Verion 4.3.1. Briefly, D-luciferin was injected into the animals intraperitoneally 10 min before the imaging procedure. Animals were maintained in general anaesthesia by isoflurane and put into the IVIS chamber for photography and detection of photon emission (large binning, F=1.2, exposure time: 3 min). The leukemia burden were measured and quantified by the same software as instructed. All the animal works were performed according to the guidelines and regulations of Animal (Scientific Procedures) Act 1986.

GST-pulldown affinity assay

5 μg of GST fusion protein was incubated with 1 mg HeLa cell lysate in NP-40 lysis buffer (150 mM NaCl, 50 mM Tris (pH 8), 5 mM EDTA, Complete Protease Inhibitor (Roche), 1% NP-40) for 2 hr at 4°C, washed with lysis buffer, eluted with SDS sample buffer & finally resolved in 10% SDS-PAGE. Protein interaction was detected by Western blotting followed by ECL chemiluminescence kit (GE Healthcare Life Science) and developed on X-ray film.

Transfection & Immunoprecipitation

Subconfluent HEK293 cells were transfected using calcium precipitation and harvested after 36-48 hrs. For generic immunoprecipitation, transfected cells were lysed in NP-40 lysis buffer (150 mM NaCl, 50 mM Tris (pH 8), 5 mM EDTA, Complete Protease Inhibitor (Roche), 0.1% -0.5% NP-40) for 30 min at 4°C, the lysates were cleared by centrifugation at 4°C. Cell lysates were incubated with the respective antibody overnight and then precipitated with protein-A/G Dynabeads (Life Technologies) at 4°C for 4 hr. For immunoprecipitation of FLAG-tagged protein, ANTI-FLAG M2 affinity agarose gel was used (Sigma Aldrich). Eluted proteins were resolved by SDS-PAGE and proteins of interest were detected with the corresponding antibody followed by Western blotting. Antibodies used for Western blotting are shown in Table 1. Densitometry analysis on immunoblot was performed with ImageJ software (Ver 1.49) according to their instructions.

Quantitative real time RT-PCR

Total RNAs extraction was performed using RNeasy kit (Qiagen) and treated with DNase (Ambion) or Nucleospin RNA extraction kit (Macherey-Nagel). cDNA was prepared using SuperscriptIII reverse transcriptase with random hexamer (Life Technology). Quantitative real time PCR was prepared with either FAST SYBR-green or Taqman probe based chemistry (Applied Biosystems) with StepOnePlus Real-Time PCR system (Applied Biosystems). RT-qPCR experiments were run in triplicate using duplicated experimental samples and analysed by $\Delta\Delta$CT method. Primer sequences are shown in Table 2.

Generation of conditional Prmt1 knockout mice

Conditional *Prmt1* knockout mouse models have been generated using targeted ES cell clones of C57BL/6 background provided by European Conditional Mouse Mutagenesis program (EUCOMM). Microinjections of the ES clones and the PCR confirmation of germline transmission were carried out by the Mammalian Genetics Unit in the Medical Research Council. The *Prmt1* allele is specifically targeted with two *loxP* sites flanking exon 5 and 6, which encode part of the methyltransferase domain. Homozygous *Prmt1*$^{flox/flox}$ mice were mated with heterozygous Rosa26-Cre-ER mice (Jackson Lab) to generate the conditional Prmt1$^{flox/flox}$ Cre-ER/+ mice. All genotypes were confirmed by PCR. Deletion of *Prmt1* in the established leukemia cell lines was induced by tamoxifen at a concentration of 50ng/ml (Sigma) after 72-96 hours.

Chromatin Immunoprecipitation

Chromatin immunoprecipitation were performed as described (Cheung et al., 2007). Briefly, cells were fixed with 1% formaldedyde for 10-20 min at room temperature and then quenched with 0.125 M glycine for 5 min. To generate DNA fragments of 0.2-1 kb, 1-10x10^6 cells were sonicated by Bioruptor Nano (Diagenode) on maximum power for 10 min with 30s on-off interval. Chromatin fragments were incubated with the antibody overnight and collected in protein-A/G dynabeads (Life Technologies). Cross-linked products were reversed by heating overnight at 65°C, incubated with RNase for 1 hr at 37°C and then treated with proteinase K at 45°C for 1 hr. Eluted DNA was purified using QIAquick PCR purification kit (Qiagen) and used for quantitative PCRs with SYBR green or Taqman according to the manufacturer's protocol. For the inducible MLL-GAS7-ER and MOZ-TIF2-ER co-immunoprecipitation experiment, 50 ng/ml tamoxifen was added to the culture medium for activation of the leukemia fusions. Antibodies used for ChIP experiment are shown in Table 1. Primer sequences are shown in Table 3.

Flow cytometry analysis and sorting

Flow cytometry analysis of murine leukemia were performed as described (Yeung and So, 2009) using c-Kit (2B8), Gr-1 (RB6-8C5), Mac-1 (M1/70), CD4 (GK1.5), CD8(53-6.7), B220 (RA3-6B2), CD45.1 (A20) and CD45.2 (104) antibodies (BioLegend) and analysed by BD LSRII (Cheung et al 2007). FACS staining of human leukemia cells were performed using CD11B (ICRF44), CD14 (HCD14), CD33 (WM53), CD34 (581), CD38 (HB-7) and CD45 (H130) antibodies (BioLegend). To investigate the leukemic mice after transplantation, cells in bone marrow, spleen and liver were analysed. The donor cells were detected by $CD45.1^{+}CD45.2^{-}$ population for murine leukemia, or CD45+CD33+ population for human leukemia. GFP-positive cells were sorted using BD FACSAria cell sorter. AnnexinV staining is performed according to manufacturer protocol (eBioscience). For cell cycle analysis, cells were fixed in cold 70% ethanol overnight, washed with PBS and treated with 100ug/ml RNAse plus propidium iodide at 37°C for 30 min. The cell cycle profiles were analysed using FlowJo software (Ver 7.6.5).

Analysis of normal hematopoiesis in vitro and vivo

c-Kit enriched HSPC were isolated from the bone marrow of SJL mice (CD45.1) and transduced with control or shKdm4c lentivirus. After 48 hours puromycin selection, 1000 transduced cells were plated into methylcellulose containing GM-CSF, SCF, IL3 and IL6 for colony forming assays and CFU assays with additional 10ng/ml erythropoietin added. The number and types of colonies were counted after 1 week. To study the effect of *Kdm4c* knockdown on normal hematopoiesis in vivo, 2x10^5 transduced SJL donor cells (CD45.1) were transplanted together with 2x10^5 recipient bone marrow mononuclear cells into lethally irradiated C57BL/6 mice (CD45.2) by tail vein injection. Peripheral blood was collected after 6 weeks by tail vein bleeding and processed for the analysis of both myeloid and lymphoid population by FACS as described in the previous section (Yeung and So, 2009).

Nitro blue tetrazolium (NBT) reduction assay

NBT reduction assay was performed to determine myeloid differentiation. NBT was added to the liquid culture at a final concentration of 0.1% and incubated at 37°C CO_2 incubator for 3hrs. The differentiated cells were indicated by the deposition of dark blue insoluble formazan (NBT positive cells) and the percentage of differentiated cells were counted under microscopy. At least 200 cells were counted in most of the cases.

RNA sequencing and GSEA analysis

For RNA sequencing and GSEA analysis, MOZ-TIF2, MLL-GAS7 and MLL-AF9 leukemic cells were derived from c-Kit positive bone marrow cells from wild type (MLL-AF9) or the conditional $Prmt1^{flox/flox}$CreER (MOZ-TIF2 and MLL-GAS7) animals by RTTA. Leukemic cells were obtained from the bone marrow of recipient animals after primary transplantation. Suppression of *Kdm4c* in MOZ-TIF2, MLL-GAS7 and MLL-AF9 leukemic cells were achieved by shRNA knockdown. Inactivation of *Prmt1* was achieved by tamoxifen treatment for 3-4 days in vitro (MOZ-TIF2 and MLL-GAS7) and compared with corresponding leukemia cells treated by vehicle control.Total RNA was isolated using mirVana miRNA extraction kit (Ambion) or Nucleopsin RNA extraction kit (Macherey-Nagel). The RNA quality was determined by Bioanalyser 2100 (Agilent)

prior to library preparation. RNA library was prepared using Truseq Stranded RNA LT kit (Illumina) and next generation sequencing was performed with HiSeq 2000/2500 (Illumina). The quality control analysis was performed on all the RNA-Seq fastq files using FastQC software (Babraham Bioinformatics). The reads were aligned using TopHat2 (Kim et al., 2013) and counted with HTSeq (Anders et al., 2015). Further statistical comparisons were performed using DESeq or DESeq2 packages (Love et al., 2014). Comparative supervised heatmaps were generated using DESeq2 package. The desktop client of gene set enrichment analysis (GSEA) (Subramanian et al., 2005) was downloaded from Broad Institute website. The weighted GSEA analysis was performed on pre-ranked gene list with the reference c2.all.v4.0.symbols.gmt [Curated] gene list and 10000 permutations. The list of pathways was reported at FDR<0.05, complied and analysed by VennPlex (Cai et al., 2013) to generate Venn diagram comparing differential alteration in signalling pathways.

Table 1. List of antibodies used for Western blotting and ChIP analysis

Antibody	Cat. Number	Use	Company
FLAG (M2)	F1804	Western blotting	Sigma
Myc (9E10)	sc-40	Western blotting	Santa Cruz
Myc (A14)	sc-789	Western blotting	Santa Cruz
Actin	I-19	Western blotting	Santa Cruz
Sam68	sc-333	Western blotting	Santa Cruz
PSF	sc-28730	Western blotting	Santa Cruz
Prmt1	ab3768	ChIP	Abcam
Prmt1	07-404	Western blotting	Millipore
GST	sc-459	Western blotting	Santa Cruz
MLL	A300-086A	ChIP	Bethyl Laboratories
H4R3me2as	39705	ChIP & Western blotting	Active Motif
Kdm4c	A300-885A	ChIP	Bethyl Laboratories
Kdm4c	NBP1-49600	ChIP	Novis Biological
H3K9me3	ab8898	ChIP & Western blotting	Abcam
H3K9ac	ab4441	ChIP	Abcam
H3K27me3	07-449	ChIP	Millipore
H3	ab1791	ChIP & Western blotting	Abcam

Table 2. RT-qPCR primer sequences

qPCR primer	Forward	Reverse	Taqman probe (5' FAM, 3' TAMRA)
Hoxa9	CCGAACACCCCGACTTCA	TTCCACGAGGCACCAAACA	TGCAGCTTCCAGTCCAAGGCGG
Meis1	CCTCGGTCAATGACGCTTTAA	TTTGAGAAATGTGAATTAGCTACTTGTACC	ACACCCCCTCTTCCCTCTCTTAGCACTGA

Prmt1	TGTTTCACAATCGGCA TCTC	CCACTCGCTGATGATGAT GT	SYBR Green
Kdm4c	AGCATGGAAAGCGAC TTGAAA	TTGTGCCGGAGAAATGC AT	CCAAGGCTTCTTCCCCAGTAGCTC CC
Myc	AGCCCCTAGTGCTGC ATGA	GCCTCTTCTCCACAGACA CC	SYBR Green
Bcat1	GGGTTCCCTACTCCAC CTCT	CGGGGCTCAGGATCACA AAG	SYBR Green
Utx	GTCGAGCCAAGGAAA TTCA	GCAGGGATTACAGTCAA CCA	CGACTTGGGCTTATGTTCAAAGTG AACA
Actb	ACCTTCTACAATGAG CTGCGT	GCTGGGGTGTTGAAGGT CT	CCCTGAACCCTAAGGCCAACCGTG A
Gapdh	Applied biosystem	Cat. No 4351309	Taqman

Table 3. Primer sequences for ChIP analysis

ChIP Primer	Forward	Reverse
Hoxa9 promoter	GAATTTGCAGGGAAAGGAAA	GGCAGGAAGAAGAAAGTGGT
Hoxa9 gene body	TTCCCTCCATTTCTTGCTTT	GCCTTTGATCACATCTCCAC
Meis1 promoter	ACTGGCTGGTTGGAGACTTT	AGCTCCCAGTTCCAGAGAAA
Meis1 gene body	AATGGGCAGGAGTTAAGGAG	TCCACGCACTGTGAATTGTA
Gapdh	AGCTGTAAGCCATGCTGTGT	GTTGTCATGGCAGCAGAAAC
Rhdopsin	CACAGGGTACTGGCTTCTTG	CGTGTGTGAAACATCCACTG
Myc promoter	CCACAGGGGCAAAGAGGATT	AGGAGTCTCTGCCGGTCTAC
Myc gene body	GGATTTCCTTTGGGCGTTGG	CTAACCGGCCGCTACATTCA

References

Anders, S., Pyl, P. T., and Huber, W. (2015). HTSeq-a Python framework to work with high-throughput sequencing data. Bioinformatics *31*, 166-169.

Becker, C. M., Wright, R. D., Satchi-Fainaro, R., Funakoshi, T., Folkman, J., Kung, A. L., and D'Amato, R. J. (2006). A novel noninvasive model of endometriosis for monitoring the efficacy of antiangiogenic therapy. Am J Pathol *168*, 2074-2084.

Cai, H., Chen, H., Yi, T., Daimon, C. M., Boyle, J. P., Peers, C., Maudsley, S., and Martin, B. (2013). VennPlex--a novel Venn diagram program for comparing and visualizing datasets with differentially regulated datapoints. PloS one *8*, e53388.

Cheung, N., Chan, L. C., Thompson, A., Cleary, M. L., and So, C. W. (2007). Protein arginine-methyltransferase-dependent oncogenesis. Nat Cell Biol *9*, 1208-1215.

Kim, D., Pertea, G., Trapnell, C., Pimentel, H., Kelley, R., and Salzberg, S. L. (2013). TopHat2: accurate alignment of transcriptomes in the presence of insertions, deletions and gene fusions. Genome biology *14*, R36.

Love, M. I., Huber, W., and Anders, S. (2014). Moderated estimation of fold change and dispersion for RNA-seq data with DESeq2. Genome biology *15*, 550.

Subramanian, A., Tamayo, P., Mootha, V. K., Mukherjee, S., Ebert, B. L., Gillette, M. A., Paulovich, A., Pomeroy, S. L., Golub, T. R., Lander, E. S., and Mesirov, J. P. (2005). Gene set enrichment analysis: a knowledge-based approach for interpreting genome-wide expression profiles. Proceedings of the National Academy of Sciences of the United States of America *102*, 15545-15550.

Yeung, J., and So, C. W. (2009). Identification and Characterization of Hematopoietic Stem and Progenitor Cell Populations in Mouse Bone Marrow by Flow Cytometry. Methods Mol Biol *538*, 301-315.

Zeisig, B. B., and So, C. W. (2009). Retroviral/Lentiviral transduction and transformation assay. Methods Mol Biol *538*, 207-229.
