# Supplementary material for: Targeting Aberrant Epigenetic Networks Mediated by PRMT1 and KDM4C in Acute Myeloid Leukemia
Source: Cancer Cell. 2016 Jan 11;29(1):32–48. doi: 10.1016/j.ccell.2015.12.007 (PMC4712026; doi:10.1016/j.ccell.2015.12.007)
Supplement: Document S2. Article plus Supplemental Information [file mmc2.pdf]

Cancer Cell

Article

Targeting Aberrant Epigenetic Networks Mediated by PRMT1 and KDM4C in Acute Myeloid Leukemia

Graphical Abstract

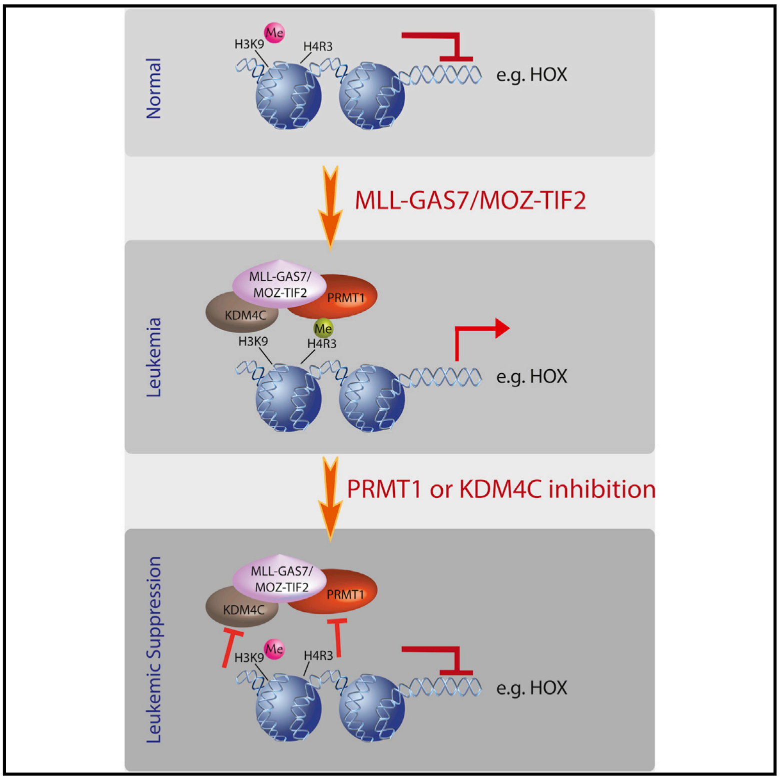

Authors

Ngai Cheung, Tsz Kan Fung,
Bernd B. Zeisig, ..., Boris Lenhard,
Li Chong Chan, Chi Wai Eric So

Correspondence

eric.so@kcl.ac.uk

In Brief

Cheung et al. show that PRMT1 is necessary but insufficient for acute myeloid leukemia induction by chimeric transcription factors, which also recruit KDM4C for epigenetic reprogramming. Pharmacological inhibition of KDM4C/PRMT1 suppresses transcription and transformation abilities of the MOZ-TIF2 and MLL fusions.

Highlights

- PRMT1 recruitment is necessary but not sufficient for transformation by AML fusions
- MLL fusions and MOZ-TIF2 recruit KDM4C to remove H3K9me3 repressive marks
- KDM4C and PRMT1 regulate aberrant epigenetic and transcriptional networks in AML
- Molecular or pharmacology inhibition of KDM4C or PRMT1 suppresses AML

Accession Numbers

E-MTAB-3322

Cheung et al., 2016, Cancer Cell *29*, 32–48
January 11, 2016 ©2016 Elsevier Inc.
http://dx.doi.org/10.1016/j.ccell.2015.12.007

Cancer Cell
Article

Targeting Aberrant Epigenetic Networks Mediated by PRMT1 and KDM4C in Acute Myeloid Leukemia

Ngai Cheung,[1] Tsz Kan Fung,[1] Bernd B. Zeisig,[1] Katie Holmes,[1] Jayant K. Rane,[1] Kerri A. Mowen,[2] Michael G. Finn,[3] Boris Lenhard,[4] Li Chong Chan,[5] and Chi Wai Eric So[1,*]
[1]Leukemia and Stem Cell Biology Group, Division of Cancer Studies, Department of Haematological Medicine, King's College London, Denmark Hill Campus, London SE5 9NU, UK
[2]Department of Chemical Physiology and Immunology & Microbial Sciences, The Scripps Research Institute, La Jolla, CA 92037, USA
[3]School of Chemistry and Biochemistry, Georgia Institute of Technology, Atlanta, GA 30332, USA
[4]Department of Molecular Sciences, Institute of Clinical Sciences, Faculty of Medicine, Imperial College London and MRC Clinical Sciences Centre, Du Cane Road, London W12 0NN, UK
[5]Department of Pathology, The University of Hong Kong, Pokfulam Road, Hong Kong
*Correspondence: eric.so@kcl.ac.uk
http://dx.doi.org/10.1016/j.ccell.2015.12.007

SUMMARY

Transcriptional deregulation plays a major role in acute myeloid leukemia, and therefore identification of epigenetic modifying enzymes essential for the maintenance of oncogenic transcription programs holds the key to better understanding of the biology and designing effective therapeutic strategies for the disease. Here we provide experimental evidence for the functional involvement and therapeutic potential of targeting PRMT1, an H4R3 methyltransferase, in various MLL and non-MLL leukemias. PRMT1 is necessary but not sufficient for leukemic transformation, which requires co-recruitment of KDM4C, an H3K9 demethylase, by chimeric transcription factors to mediate epigenetic reprogramming. Pharmacological inhibition of KDM4C/PRMT1 suppresses transcription and transformation ability of MLL fusions and MOZ-TIF2, revealing a tractable aberrant epigenetic circuitry mediated by KDM4C and PRMT1 in acute leukemia.

INTRODUCTION

Human leukemia is characterized by the prevalence of recurrent chromosomal translocations, resulting in the generation of chimeric fusion proteins with aberrant oncogenic activities (Look, 1997). Successful therapeutic exploitation of BCR-ABL fusion in chronic myeloid leukemia (CML) by the small molecular inhibitor imatinib has become the paradigm of targeted therapy (Kantarjian et al., 2002). In contrast, there has been very little progress in targeting classically intractable oncogenic transcription factors, which are the common drivers for many other malignancies including acute myeloid leukemia (AML) (Zeisig et al., 2012). With the exception of acute promyelocytic leukemia (APL) for which targeted therapy has been developed, transforming it from a highly fatal disease to a manageable condition (Arteaga et al., 2015; Wang and Chen, 2008), all AML patients still receive the same chemotherapy treatment developed more than half a century ago, which only induces long-term complete remission in less than 40% of young patients and is generally too toxic to use in patients aged older than 60 years (Zeisig et al., 2012). Therefore, there is an urgent need to understand the underlying transformation mechanisms and develop better therapeutic strategies for AML.

In contrast to kinases that already have functional enzymatic activities, transcription factors need to work in tandem with other co-factors to orchestrate an array of epigenetic modifications for regulating gene expression. Among these factors are protein methyltransferases (PMTs), consisting of lysine methyltransferases (KMTs) and arginine methyltransferases (PRMTs), which have recently taken the center stage as key players in

Significance

While the recent launch of phase I clinical trials with protein-methyltransferase (PMT) inhibitors has ignited the enthusiasm for targeting oncogenic transcription factors, our understanding of the functions of PMTs in cancer development is still in its infancy. This has limited the potential for exploiting this group of promising targets. Here, we reveal critical functions and preclinical in vivo evidence for targeting a second class of PMTs, PRMT, and histone demethylases (KDMs) in cancer. PRMT1 is necessary but not sufficient for leukemia induction by chimeric transcription factors, which also recruit KDM4C, for epigenetic reprogramming. Genetic or pharmacological inhibition of KDM4C/PRMT1 suppresses transcription and transformation abilities of the MOZ-TIF2 and MLL fusions, providing druggable therapeutic targets and molecular insights for the development of epigenetic therapy.

 ©2016 Elsevier Inc.

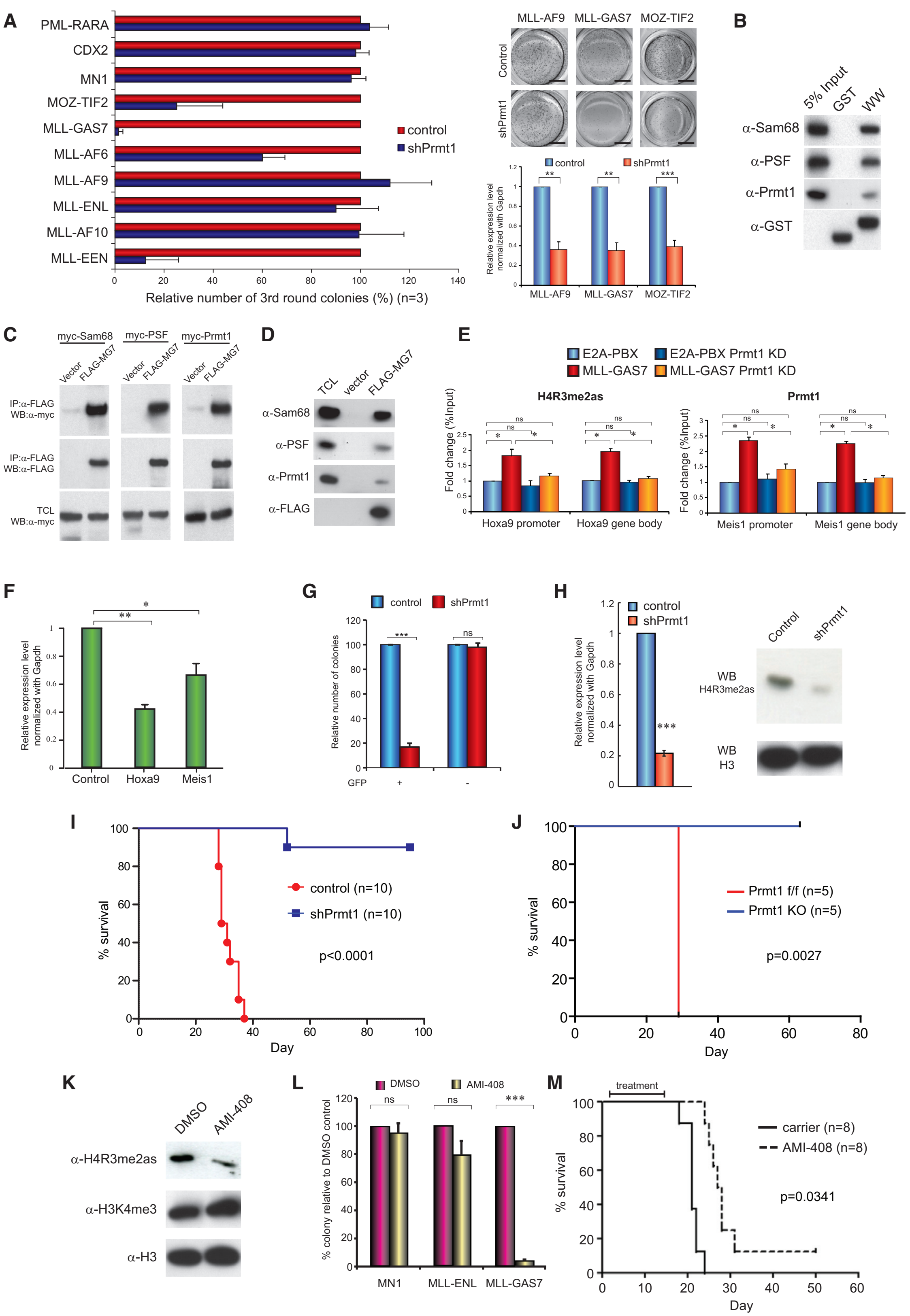

Figure 1. Targeting of Prmt1 Suppresses MLL-GAS7 Leukemia

(A) Effect of Prmt1 knockdown on serial replating of transformed cells induced by various leukemic fusions. qRT-PCR analysis of *Prmt1* knockdown in leukemic cells. Scale bars represent 0.5 cm.

(legend continued on next page)

transcription regulation during both normal and disease development (Abdel-Wahab and Levine, 2013; Cheung and So, 2011; Kouzarides, 2007). The involvements and therapeutic potential of targeting PMTs in human cancer were initially illustrated in MLL leukemia where the recruitments of DOT1L by MLL-AF10 (Okada et al., 2005) and PRMT1 by MLL-EEN (Cheung et al., 2007) were required for transcriptional deregulation and cellular transformation. Since then, additional members of the PMT family have been reported to be involved in different cancers (Campbell and Tummino, 2014; Cheung and So, 2011; Shih et al., 2012). The promise of targeting PMTs for cancer treatment has been highlighted by the successful development of chemical inhibitors against DOT1L for MLL leukemia (Daigle et al., 2011) and EZH2 for B-cell lymphoma carrying EZH2-activating mutations (Knutson et al., 2012; McCabe et al., 2012); these are now entering phase I clinical trials.

Despite the success in development of inhibitors, the field is still in its infancy and the involvements of PMTs, in particular PRMTs, in other leukemias are still largely unexplored. More importantly, we have very little knowledge about the mechanisms and molecular networks that underpin the oncogenic functions mediated by these individual PMTs. The discovery of JmjC domain-containing lysine demethylases (KDMs) has provided unique insights into dynamic regulation of histone methylation for gene regulation. KDMs can work together with specific KMTs to remove the opposing methylation marks to reinforce particular epigenetic programs for gene expression (Cloos et al., 2008). Consistent with this, a recent study reported an important role of KDM5B in suppressing the epigenetic program and function of leukemic stem cells, while its therapeutic value has yet to be demonstrated by pharmacological means (Wong et al., 2015). On the other hand, members of KDMs including JHDM1B (He et al., 2011) and JMJD1C (Sroczynska et al., 2014) have been shown to be required for leukemic transformation. In spite of these interesting observations indicating important and contrasting roles of KDMs in leukemogenesis, very little is known about their actual functions and underlying mechanisms. It is not clear whether and how the dynamic functional interplay between PMTs and KDMs takes part in regulating this critical process. More importantly, in contrast to the recent demonstration of specific in vivo efficacy of poly(ADP ribose) polymerase (PARP) inhibitors in certain subtypes of AML (Esposito et al., 2015), there have been no in vivo pharmacological inhibitor data showing the potential value of targeting KDMs for leukemia suppression. Together, these have significantly hindered the potential translation of these findings into the relevant clinical utility. Therefore, elucidation of the functional and mechanistic involvement of histone methylation machinery will shed light on the ongoing efforts to understand the mechanisms underlying the roles of these different epigenetic modifying enzymes in cancer biology, and the development of effective therapeutic strategies targeting the associated oncogenic transcription factors in human cancer.

RESULTS

Identification of Prmt1-Dependent Leukemia Fusion Proteins

To define the functional involvement of Prmt1 in leukemias, we performed a systematic functional screen by retroviral transduction and transformation assay (RTTA) using validated Prmt1 small hairpin RNAs (shRNAs) on more than ten different MLL and non-MLL oncogenic transcription factors. As a control, downregulation of Prmt1 suppressed MLL-EEN-mediated transformation of primary c-kit enriched hematopoietic stem/progenitor cells (HSPCs) (Cheung et al., 2007). While transformation mediated by most of the other MLL fusions were not affected by Prmt1 knockdown, MLL-GAS7 (So et al., 2003a) and MOZ-TIF2 (Huntly et al., 2004) exhibited high degrees of Prmt1 dependence, resulting in a significant suppression of colony formation (Figure 1A). These data were reproduced using an independent Prmt1 shRNA (shPrmt1#2) (Figures S1A and S1B). In line with the RTTA data, suppression of Prmt1 resulted in an increased differentiation (Figure S1C), cell-cycle arrest particularly at G1 checkpoint (Figure S1D), and an enhanced apoptosis (Figure S1E) in both MLL-GAS7 and MOZ-TIF2 transformed cells. Discovery of additional Prmt1-dependent oncogenic fusions prompted us to speculate whether they utilized similar epigenetic machinery and, hence, recruitment of communal transcription complexes to transform HSPC. Consistent with this idea, GAS7 WW domain has been proposed to interact with Sam68 and PSF (Ingham et al., 2005), which are the key components of MLL-EEN/Prmt1 transcriptional complex (Cheung et al., 2007). To this end, both in vitro glutathione S-transferase (GST)

(B) GST pull-down assays to show the interaction of GAS7 WW domain (WW) with Sam68, PSF, and Prmt1 in vitro.
(C and D) Co-immunoprecipitation of FLAG-MLL-GAS7 with myc-tagged Prmt1, Sam68, and PSF (C) and endogenous Sam68, Prmt1, and PSF (D).
(E) ChIP analysis on the effect of Prmt1 knockdown on H4R3me2as mark and Prmt1 binding in *Hoxa9* promoter and gene body region of MLL-GAS7 and E2A-PBX.
(F) qRT-PCR analysis on *Hoxa9* and *Meis1* expression in MLL-GAS7 after Prmt1 knockdown.
(G) MLL-GAS7 leukemic cells transduced with control or shPrmt1 lentivirus expressing GFP markers. Transduced populations were sorted based on GFP expression and plated into methylcellulose to study colony-forming ability.
(H) *Prmt1* knockdown was validated by qRT-PCR, and its effect on H4R3me2as marks was analyzed by western blot with histone H3 as the loading control.
(I) Kaplan-Meier survival analysis of the effect of Prmt1 knockdown on MLL-GAS7 leukemogenesis (log-rank test $p < 0.0001$). Median disease latency: control, 30 days; shPrmt1, undefined.
(J) Kaplan-Meier survival analysis of mice transplanted with wild type (WT) or *Prmt1* knockout (KO) MLL-GAS7 leukemia cells (log-rank test $p = 0.0027$). Median disease latency: WT, 29 days; *Prmt1* KO, undefined.
(K) Western blot analysis of H4R3me2as and H3K4me3 after AMI-408 treatment with histone H3 control for histone loading.
(L) Effect of AMI-408 on colony formation of murine leukemia cell lines.
(M) Kaplan-Meier survival analysis on the effect of AMI-408 treatment on MLL-GAS7 leukemogenesis (log-rank test $p = 0.0341$). Median disease latency: control, 21 days; AMI-408, 27.5 days.
All data shown are mean and SD ($n = 3$) unless otherwise specified. See also Figure S1. For all figures, asterisks indicate $^{*}p < 0.05$, $^{**}p < 0.01$, and $^{***}p < 0.001$; ns, not significant.

pull-down using GAS7 WW domain (Figure 1B) and immunoprecipitation assay by co-expression of candidate proteins (Figure 1C) had successfully demonstrated the ability of GAS7 to recruit Sam68, PSF, and Prmt1. In addition, we also confirmed in vivo interactions between MLL-GAS7 and the endogenous Sam68, PSF, and Prmt1 by immunoprecipitation experiments using antibodies specific to the endogenous proteins (Figure 1D). To further evaluate the in vivo interaction in the context of chromatin and their epigenetic functions, we deployed chromatin immunoprecipitation (ChIP) to demonstrate the specific binding of Prmt1 and the associated asymmetric H4R3 dimethylation (H4R3me2as) activation mark to the downstream targets of MLL fusion, *Hoxa9* (Figure 1E). As a result, we were able to detect significant enhancements of Prmt1 binding and the associated H4R3me2as marks in both the promoter and gene body regions of *Hoxa9* in MLL-GAS7 transformed cells but not in the E2A-PBX control (Figure 1E). Conversely, loss of Prmt1 through shRNA-mediated knockdown resulted in a reduction of H4R3me2as mark (Figure 1E) and the suppressed expression of MLL downstream targets (Figure 1F), confirming a critical function of Prmt1 in MLL-GAS7-mediated transcription deregulation.

PRMT1 Is Required for Maintenance of MLL-GAS7 Leukemia

To investigate whether Prmt1 is required for not only initiation (Figure 1A) but also maintenance of the leukemic transformation, we transduced MLL-GAS7 full-blown leukemia cells from primary transplanted mice (So et al., 2003b) with lentivirus co-expressing a GFP marker and Prmt1 shRNA or a scramble control for in vitro and in vivo transformation assays. In contrast to GFP-negative cells, which did not show any significant difference in colony-forming ability regardless of shRNA constructs being used, GFP-positive cells carrying shPrmt1 had a severely compromised colony-forming ability compared with their scramble control (Figure 1G). The effectiveness of Prmt1 knockdown was confirmed by both qRT-PCR on *Prmt1* mRNA and immunoblot on the associated H4R3me2as mark (Figure 1H). To assess the in vivo leukemogenic function of Prmt1, we transplanted MLL-GAS7 cells into syngeneic mice for disease development. Cohorts transplanted with Prmt1 knockdown leukemia cells exhibited increased disease latency and a reduced penetrance compared with the scramble control (log-rank test $p < 0.0001$) (Figures 1I, S1F, and S1G). Interestingly, the only mouse transplanted with Prmt1 knockdown cells that succumbed to leukemia re-expressed high levels of *Prmt1* and *Hoxa9* (Figure S1H), suggesting a high selective pressure against Prmt1 knockdown for leukemia development. To further address this point, we developed a *Prmt1* Cre-ER conditional knockout mouse where exons 5–6 spanning the catalytic domain could be conditionally deleted upon tamoxifen treatment, resulting in a truncated protein. Using primary c-kit^{+} HSPCs from this *Prmt1$^{flox/flox}$* Cre-ER mouse for RTTA, we observed an even more prominent suppression of MLL-GAS7 transformed cells both in vitro (Figures S1I and S1J) and in vivo (Figure 1J) whereby none of the mice developed leukemia upon Prmt1 deletion. Together, these independent approaches confirm a critical function of Prmt1 in both leukemia initiation and maintenance.

Pharmacological Inhibition of PRMT1 Suppresses AML In Vivo

To further demonstrate the therapeutic potential of targeting Prmt1, we examined the effect of an early-phase PRMT1 inhibitor, AMI-408 (Bonham et al., 2010) (Figure S1K) on the suppression of MLL-GAS7 mediated leukemogenesis. Consistently, treatment of MLL-GAS7 leukemia cells with AMI-408 resulted in the reduction of H4R3me2as mark (Figure 1K) and reduced colony-forming ability (Figure 1L). Importantly, in vivo administration of AMI-408 to mice transplanted with pretreated MLL-GAS7 leukemia cells significantly extended the survival and reduced disease penetrance compared with the carrier control ($p = 0.0341$) (Figure 1L), revealing the therapeutic potential of targeting Prmt1 by a small-molecule inhibitor.

Recruitment of PRMT1 Is Indispensable for MOZ-TIF2-Mediated Leukemogenesis

To further understand the functional role of Prmt1 in other leukemia subtypes, we sought to dissect the roles of Prmt1 in MOZ-TIF2-mediated transformation. Given that aberrant recruitment of Prmt1 appears to be a common feature shared by different MLL fusions, we intuitively examined the possible recruitment of Prmt1 by MOZ-TIF2. Using immunoprecipitation assays, we were able to show the specific interaction of MOZ-TIF2 with both ectopically expressed and endogenous Prmt1 (Figure 2A). To further demonstrate the in vivo functional interaction in MOZ-TIF2 leukemic cells, ChIP analysis revealed specific recruitment of Prmt1 and a high level of H4R3me2as mark on the downstream targets of MOZ-TIF2, *Hoxa9* loci (Katsumoto et al., 2006; Kvinlaug et al., 2011), implicating a mechanistic similarity among those PRMT1-dependent leukemic fusions (Figure 2B). To gain insights into this Prmt1 interaction, we prepared various MOZ-TIF2 deletion mutants, which were used to map the Prmt1 interaction domain by co-immunoprecipitation assays. As a result, MOZ 5′ was sufficient to recruit Prmt1, and deletion of its N-terminal 310 amino acids (containing an N-terminal domain, H15 and PHD) completely abolished the interaction (Figures 2C and 2D). Further progressive deletion analysis refined the first 79 amino acids of the N-terminal domain but not H15 and PHD of the fusion as the minimal interaction domain required for Prmt1 recruitment, and conferring its epigenetic mark (Figures 2C–2E).

To examine the significance of Prmt1 interaction in leukemic transformation, we performed structure-function analysis using the corresponding MOZ-TIF2 deletion mutants to evaluate their transformation ability (Figures 2D and S2A). An internal deletion of H15 or PHD did not compromise cellular transformation, whereas all the mutants with a deletion of the N-terminal Prmt1 interaction domain failed to transform HSPC (Figure 2D), consistent with a critical function of Prmt1 recruitment for MOZ-TIF2 transformation. To further assess the requirement of Prmt1 for leukemia maintenance, we transduced MOZ-TIF2 leukemic cells carrying a ubiquitin C promoter (UbC)-driven luciferase reporter (Becker et al., 2006) harvested from primary leukemia mice with either shPrmt1 or scramble control lentivirus prior to transplantation into syngeneic mice for leukemia development. As a result, in vivo imaging demonstrated reduced leukemia burdens for mice carrying Prmt1 knockdown leukemic cells (Figure 2F). Prmt1 knockdown also significantly extended the latency and reduced penetrance of the disease compared with the control

CellPress

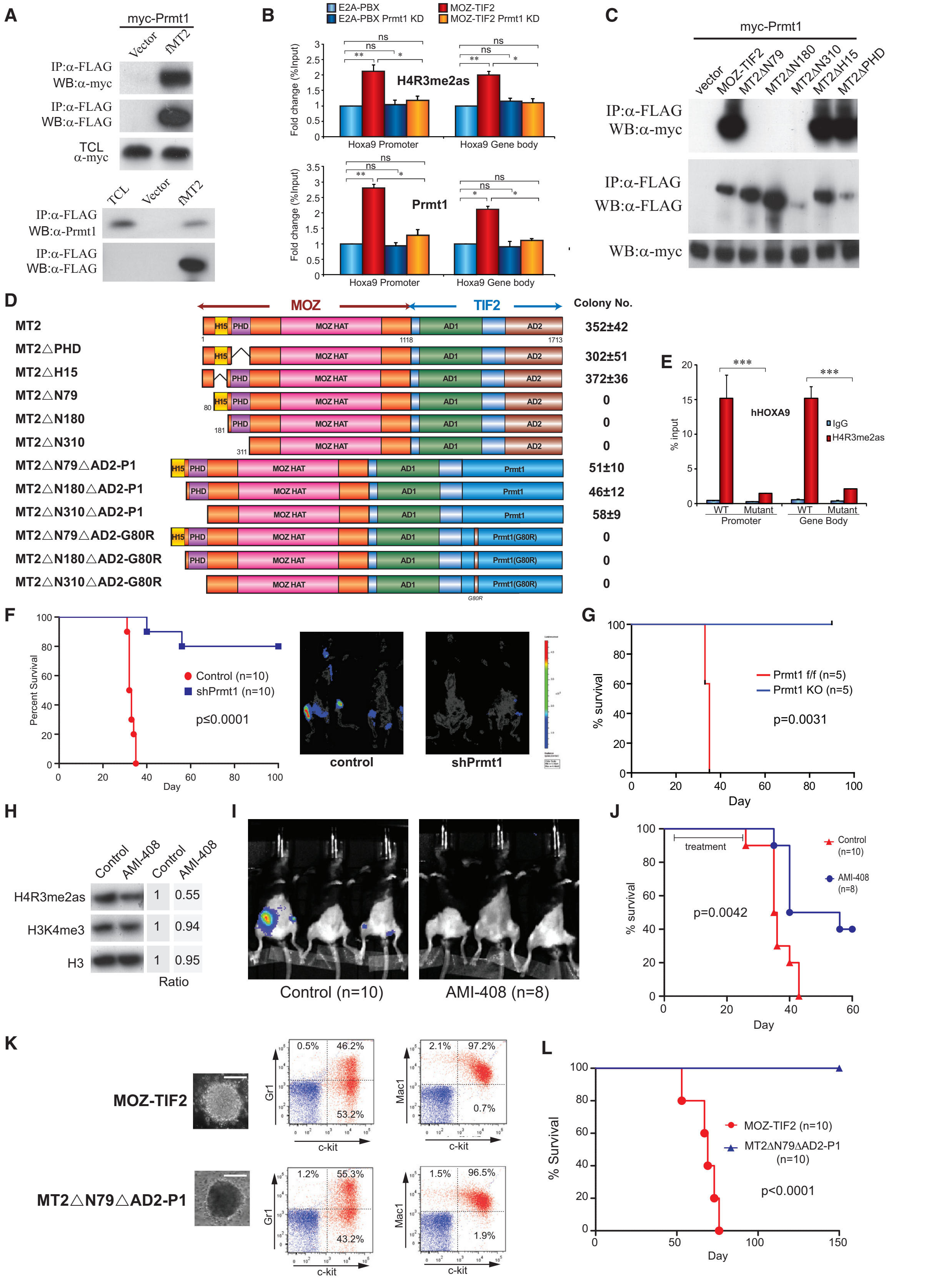

(legend on next page)

cohort (log-rank test $p < 0.0001$) (Figures 2F, S2B, and S2C). Similar to the MLL-GAS7 studies, there was strong pressure on select leukemia clones to escape from Prmt1 knockdown as indicated by their re-expression of *Prmt1*, and *Hoxa9* in the leukemic mice received MOZ-TIF2 Prmt1 knockdown cells (Figure S2B). Consistently, an irreversible inactivation of *Prmt1* in MOZ-TIF2 transformed cells using conditional knockout approach in RTTA not only significantly suppressed their in vitro colony-forming ability (Figures S1I and S1J) but also abolished their in vivo leukemogenic potentials (Figure 2G). To further demonstrate the in vivo therapeutic potentials of targeting Prmt1 in the clinically relevant setting, we transplanted MOZ-TIF2 leukemia cells carrying UbC-luciferase reporter without any pretreatment into syngeneic mice and then subjected them to AMI-408 treatment. As expected, in vivo AMI-408 treatment suppressed H4R3me2as mark in MOZ-TIF2 leukemia cells (Figure 2H). More importantly, AMI-408 significantly reduced the tumor burdens (Figure 2I) and extended the leukemia latency ($p = 0.0042$) (Figure 2J). Although AMI-408 is an early-phase PRMT1 inhibitor that clearly requires further optimization to improve its potency, these results provide the proof-of-principle experimental data showing in vivo efficacy of pharmacological targeting of Prmt1 for leukemia suppression.

Aberrant Recruitment of PRMT1 Is Necessary but Not Sufficient for Induction of AML In Vivo

While structure-function analysis, shRNA-mediated knockdown, genetic knockout, and pharmacological inhibition experiments clearly indicate an essential role of Prmt1 in MOZ-TIF2 leukemia, it remains to be determined whether Prmt1 recruitment per se is sufficient and the sole function of the N-terminal minimal transformation domain required for MOZ-TIF2-mediated leukemogenesis. To this end, Prmt1 was covalently linked to transformation-defective MOZ-TIF2 N-terminal truncation mutants (ΔN79, ΔN180, and Δ310) to examine whether Prmt1 swapping is sufficient to resurrect their transformation activity. As a result, direct fusion of Prmt1 to those transformation-defective N-terminal deletion mutants was able to confer serial replating ability and established primary transformed cell lines, albeit the number of third-round colony was reduced (Figure 2D). In contrast, covalent fusion of Prmt1 catalytically inactive mutant carrying a single point mutation in the enzymatic domain failed to resurrect the transformation ability of any of these N-terminal deletion MOZ-TIF2 mutants (Figure 2D), despite their expression at a comparable level (Figure S2A). Immunophenotypic analysis of the MOZ-TIF2-Prmt1 transformed cells confirmed the phenotypes of early myeloid progenitors (c-kit, Gr1, and Mac1), which were similar to wild-type (WT) MOZ-TIF2 leukemic cells (Figure 2K). We then tested whether Prmt1 swapping could also rescue leukemogenesis in vivo by transplanting primary HSPC immortalized with WT MOZ-TIF2 or MOZ-TIF2-Prmt1 (MT2ΔN79ΔAD2-Prmt1) into syngeneic mice for leukemia development. Surprisingly, only mice transplanted with WT MOZ-TIF2 induced leukemia in vivo with a median disease latency of 61 days, whereas no leukemia was found in the cohort injected with MOZ-TIF2-Prmt1 fusion immortalized cells (Figure 2L), suggesting that additional hitherto unidentified molecules may also be recruited by the N-terminal transformation domain and are required for leukemogenesis.

MLL Fusions and MOZ-TIF2 Recruit KDM4C to Control H3K9me3 Status of Their Target Genes

In addition to PMTs, KDMs frequently act in tandem with PMTs for regulating gene expression critical for normal and disease development (Cheung and So, 2011). This prompted us to explore whether MOZ-TIF2 could interact and collaborate with other KDMs to induce oncogenic transcriptional programs. To this end, we performed a systematic biochemical screen to identify potential KDMs that may interact with MOZ-TIF2. Co-immunoprecipitation using MOZ-TIF2 and different KDMs revealed a highly specific interaction with KDM4C but not any other tested demethylases (Figure 3A). Reciprocal co-immunoprecipitation experiments were then performed to map the KDM4C interaction domain within MOZ-TIF2 using different deletion constructs comprising the MOZ and TIF moiety of the fusion (Figure 3B). KDM4C interaction was maintained by MOZ 5′ but not TIF 3′ moiety. Further deletion analysis refined the minimal KDM4C interaction domain within the first N-terminal 79 amino acids of MOZ moiety, which overlaps with the PRMT1 interaction and the aforementioned minimal N-terminal transformation domain.

Figure 2. Recruitment of Prmt1 by MOZ-TIF2 Is Necessary but Not Sufficient for HSPC Transformation

(A) Co-immunoprecipitation of FLAG-MOZ-TIF2 (fMT2) with myc-Prmt1 (upper) and endogenous Prmt1 (lower).

(B) ChIP analysis of H4R3me2as and Prmt1 localization on *Hoxa9* promoter and gene body in MOZ-TIF2 after Prmt1 knockdown (KD).

(C) Co-immunoprecipitation of different FLAG-MOZ-TIF2 deletion mutants (indicated in D) and myc-Prmt1.

(D) RTTA to study the effect of N-terminal deletion mutants, active and inactive Prmt1 (P1) rescue fusions of MOZ-TIF2 on leukemic transformation.

(E) ChIP analysis of H4R3me2as mark on *HOXA9* loci in HEK293 cells transfected with WT MOZ-TIF2 or ΔN79.

(F) Kaplan-Meier survival analysis of the effect of *Prmt1* knockdown on MOZ-TIF2-mediated leukemogenesis (log-rank test $p \leq 0.0001$). Bioluminescence imaging was performed at 21 days after transplantation. Median disease latency: control, 33 days; shPrmt1, undefined.

(G) Kaplan-Meier survival analysis of mice transplanted with WT or *Prmt1* KO MOZ-TIF2 leukemia cells (log-rank test $p = 0.0031$). Median disease latency: WT, 35 days; *Prmt1* KO, undefined.

(H) Western blot analysis on the effect of AMI-408 on H4R3me2as, H3K4me3, and the loading control histone H3. Band intensity ratio was determined by densitometry and normalized to vehicle control.

(I) Bioluminescence imaging of mice transplanted with MOZ-TIF2-luciferase leukemic cells 3 weeks after AMI-408 or carrier treatment.

(J) Kaplan-Meier survival analysis of AMI-408 and control treatment on MOZ-TIF2 leukemogenesis (log-rank test $p = 0.0042$). Median disease latency: control, 35.5 days; AMI-408, 48 days.

(K) Morphology of third-round colony of HSPC transformed by MOZ-TIF2 and its Prmt1 rescue fusion. FACS analysis of the transformed cells stained with c-kit, Gr1, and Mac1. Scale bars represent 50 μm.

(L) Kaplan-Meier survival analysis of mice transplanted with MOZ-TIF2 or MT2ΔN79ΔAD2-P1 transformed cells (log-rank test $p < 0.0001$). Median disease latency: MOZ-TIF2, 69 days; MT2ΔN79ΔAD2-P1, undefined.

All data shown are mean and SD (n = 3) unless otherwise specified. See also Figure S2.

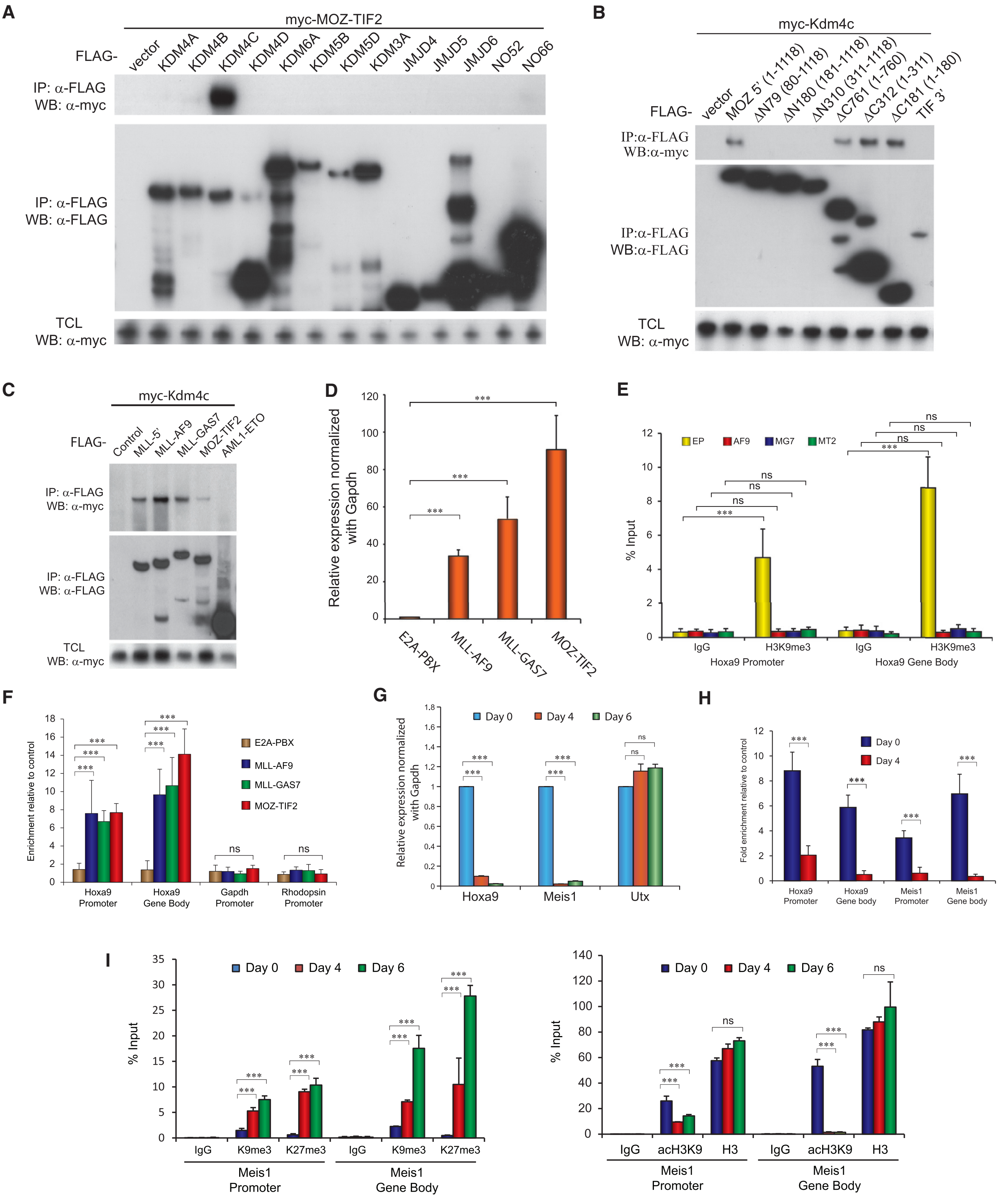

Figure 3. MOZ-TIF2 and MLL Fusions Recruits Kdm4c to Regulate H3K9 Methylation Status of Their Target Genes

(A–C) Co-immunoprecipitation of FLAG-KDMs with myc-MOZ-TIF2 (A); FLAG-MOZ deletion constructs and myc-KDM4C (B); FLAG-MLL fusions, MOZ-TIF2, and AML1-ETO with myc-KDM4C (C).

(D) qRT-PCR analysis of *Hoxa9* expression in murine leukemia cell lines.

(E) ChIP analysis of the H3K9me3 methylation on *Hoxa9* loci of E2A-PBX (EP), MLL-AF9 (AF9), MLL-GAS7 (MG7), and MOZ-TIF2 (MT2).

(legend continued on next page)

This leads to the hypothesis that KDM4C may represent the additional epigenetic regulator that can cooperate with PRMT1 in mediating transcriptional deregulation and acute leukemogenesis. To this end, we further explored the role of KDM4C in other PRMT1-dependent MLL oncoproteins, and were able to demonstrate a strong interaction between KDM4C and MLL-GAS7 (Figure 3C). Biochemical mapping revealed the interaction domain located at MLL 5′, which is present in all MLL fusions. Consistently, KDM4C was recruited by other MLL fusions such as MLL-AF9 but not the non-MLL fusion control AML1-ETO (Figure 3C).

To further demonstrate these interactions in vivo, we carried out transcriptional and epigenetic analyses using HSPC transformed by MLL fusions, MOZ-TIF2, or E2A-PBX control. KDM4C is a lysine demethylase that catalyzes the specific removal of the repressive H3K9 methylation marks and may be required to maintain its target genes in an open chromatin configuration for gene expression. In line with this hypothesis, the level of H3K9me3 mark was inversely correlated with the expression status of *Hoxa9*, a downstream transcriptional target of both MLL fusions and MOZ-TIF2 (Figures 3D and 3E). In contrast to the control E2A-PBX transformed cells, H3K9me3 mark on *Hoxa9* loci was significantly lower in HSPC transformed by MLL fusions or MOZ-TIF2 that recruited endogenous Kdm4c to the promoter (Figures 3E and 3F). In addition, loss of H3K9me3 was concomitant with the increased H3K9 acetylation in MLL fusion or MOZ-TIF2 transformed cells (Figure S3A). To gain further insights into the dynamic interplay of H3K9 methylation and KDM4C in transcriptional regulation, we employed an inducible MLL-AF9-ER transformed primary cell line, in which MLL-AF9 was fused to ER, allowing its activity to be regulated by tamoxifen. Upon tamoxifen withdrawal, we detected significantly reduced expression of MLL downstream targets including *Hoxa9* and *Meis1* (Figure 3G) and a marked reduction of MLL fusion binding to the *Hoxa9* and *Meis1* promoters (Figure S3B). Lower MLL fusion binding also led to a concomitant reduced recruitment of endogenous Kdm4c on *Hoxa9* and *Meis1* loci (Figure 3H). Consistently, loss of Kdm4c binding was further accompanied by reduction of H3K9 acetylation and the accumulation of repressive H3K9me3 and H3K27me3, indicating repressive transcription complex domination in the absence of KDM4C (Figure 3I). Together, the results indicate that the dynamics of H3K9 methylation and acetylation is tightly regulated by the recruitment of KDM4C by the leukemic fusions.

Aberrant Transcriptional Networks Co-regulated by KDM4C and PRMT1 in MLL and MOZ-TIF2 Leukemia

To investigate the transcriptional functions and potential crosstalk between KDM4C and PRMT1 in acute leukemogenesis, we performed global transcriptional analyses by RNA sequencing (RNA-seq) in both MOZ-TIF2 and MLL-GAS7 transformed cells in the presence or absence of Kdm4c or Prmt1 using shRNA knockdown and conditional knockout approach, respectively (Figures S4A and S4B). Differential expression gene lists from two biological replicates were used to generate heatmaps, which revealed highly similar and overlapping gene expression signatures associated with the loss of Kdm4c and Prmt1 ($p = 1.97 \times 10^{-54}$ for MOZ-TIF2 and 5.71×10^{-92} for MLL-GAS7) (Figure 4A), consistent with their critical functions in mediating transcriptional programs initiated by the fusions. To gain further insights into the molecular pathways co-regulated by these two epigenetic modifying enzymes, we deployed gene set enrichment analysis (GSEA) to identify potential pathways perturbed by the loss of function of Kdm4c and Prmt1. Consistently, a very large number of overlapping pathways were perturbed by the loss of function of Prmt1 and Kdm4c (Figure 4B). As transcriptional activators, about 37% (201 of 548) of the pathways activated by Kdm4c were also regulated by Prmt1 in MOZ-TIF2 transformed cells. Even more strikingly, 90% (201 of 222) of the pathways downregulated by Prmt1 inhibition were also suppressed upon Kdm4c knockdown, suggesting a strong transcriptional co-regulation mediated by these two different classes of epigenetic modifying enzymes. We also observed very similar transcriptional co-regulations between Prmt1 and Kdm4c in MLL-GAS7 transformed cells, in which 52% (314 of 603) of Prmt1 activated pathways and 74% (314 of 427) of Kdm4c activated pathways were co-regulated by each other (Figure 4B). These transcriptional analysis results are also consistent with biochemical study showing that interaction between Prmt1 and Kdm4c was largely dependent on MLL-GAS7 and MOZ-TIF2 (Figure S4C), supporting the hypothesis that these two epigenetic modifying enzymes are recruited by the leukemic fusions to execute aberrant transcription programs.

KDM4C Is Required for Maintenance of Transcriptional Programs by MLL Fusions

Since MLL-AF9 recruits only Kdm4c but not Prmt1, we examined whether suppression of Kdm4c was sufficient to interrupt the oncogenic transcription programs maintained by the Prmt1-independent MLL fusion. Kdm4c knockdown in MLL-AF9 transformed cells resulted in transcriptional signatures similar to those in MLL-GAS7 transformed cells upon Prmt1/Kdm4c suppression (Figures 4C and 4D). More than 35% (797 of 2,217, $p = 3.28 \times 10^{-57}$) of the genes and 60% (135 of 215) of the pathways significantly downregulated in MLL-AF9 transformed cells upon Kdm4c knockdown overlapped with those in MLL-GAS7, indicating a common requirement of Kdm4c in the maintenance of transcription programs in different MLL leukemias. Interestingly, there was a number of pathways commonly regulated by both Prmt1 and Kdm4c in these fusions: these include the Myc pathway and embryonic stem cell program (Figures 4E and 4F), which are important for MLL leukemia (Dawson et al., 2011; Somervaille et al., 2009) and other AML (Zuber et al., 2011). To validate some of the findings from RNA-seq, qRT-PCR

(F) ChIP analysis of Kdm4c localization at the promoter and gene body region of *Hoxa9* in murine leukemia cell lines.

(G) qRT-PCR analysis of *Hoxa9*, *Meis1*, and *Utx* expression 4 days after tamoxifen withdrawal in the inducible MLL-AF9-ER cells.

(H) ChIP analysis of Kdm4c at the promoter and gene body regions of *Hoxa9* and *Meis1* loci of MLL-AF9-ER 4 days after tamoxifen withdrawal.

(I) ChIP analysis of H3K9me3, H3K27me3 (left) and acH3K9 and H3 (right) on the promoter and gene body regions of *Meis1* 4 and 6 days after tamoxifen withdrawal.

All data shown are mean and SD (n = 3) unless otherwise specified. See also Figure S3.

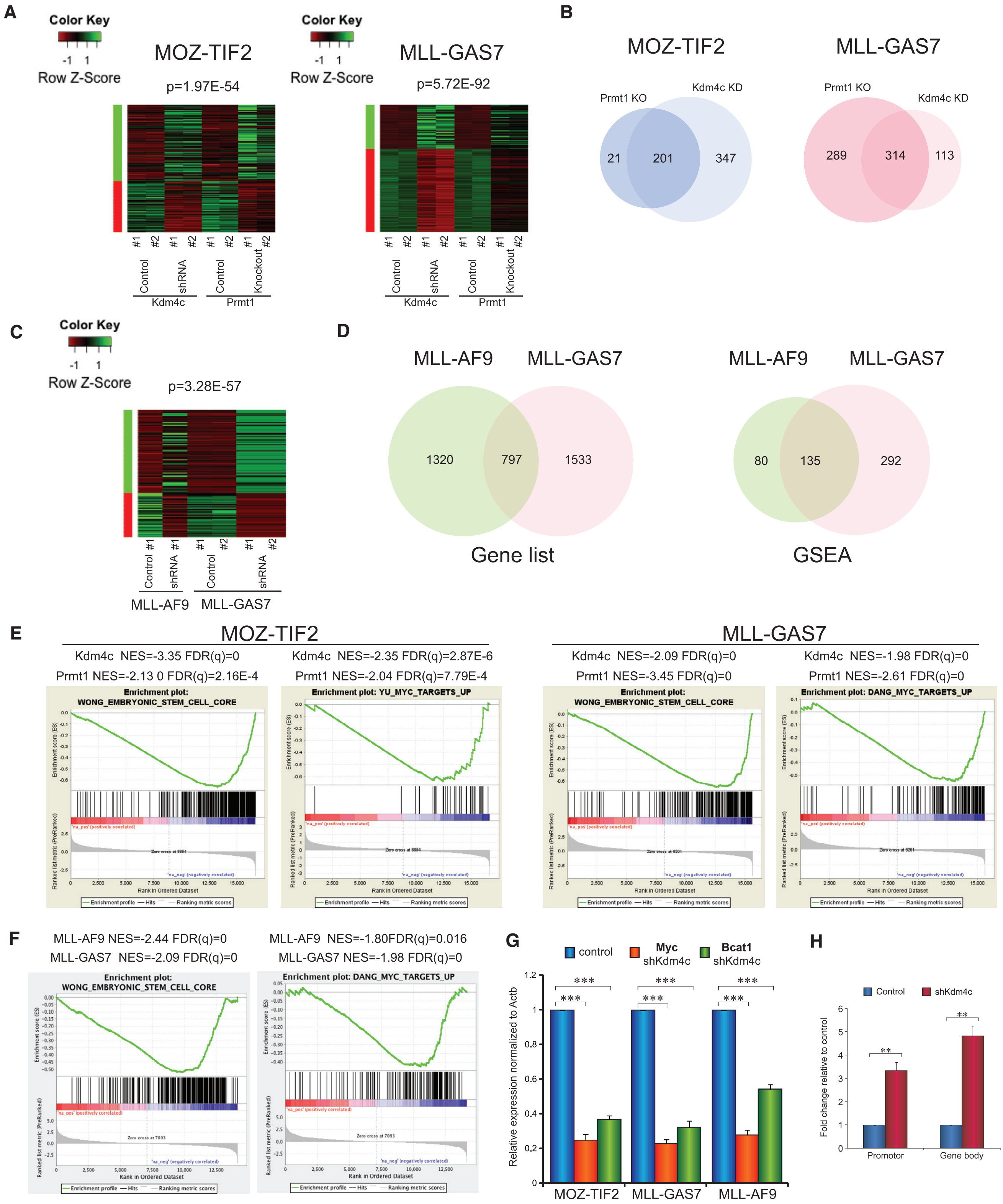

Figure 4. RNA-Seq Reveals Overlapping Pathways Targeted by Both Prmt1 and Kdm4c

(A) Heatmap analysis of gene expression profile in MOZ-TIF2 and MLL-GAS7 leukemic cells after Prmt1knockout and Kdm4c knockdown.

(B) Venn diagram showing the common downregulated pathways after the loss of function of Prmt1 and Kdm4c (overlapped) identified by GSEA.

(C) Heatmap analysis of gene expression signature perturbed in both MLL-AF9 and MLL-GAS7 after Kdm4c knockdown.

(legend continued on next page)

experiments confirmed that Kdm4c knockdown resulted in the suppression of expression of *Myc* as well as its target *Bcat1* in MLL-GAS7, MLL-AF9, and MOZ-TIF2 leukemia cells (Figure 4G). Consistently, Kdm4c knockdown led to the upregulation of H3K9me3 marks on *Myc* loci in MLL transformed cells (Figure 4H), indicating a critical function of KDM4C in regulating oncogenic transcriptional networks.

KDM4C Is Essential for Initiation and Maintenance of MLL and MOZ-TIF2 Leukemia

We next investigated the functional requirement of Kdm4c for leukemic transformation. Suppression of Kdm4c by two independent shRNAs (Figure S5A) resulted in a similar and significant reduction of the serial replating ability of MLL fusions and MOZ-TIF2 transformed cells compared with their relatively moderate effect on E2A-HLF transformed cells (Figure 5A). Inhibition of Kdm4c led to increased differentiation (Figure 5B), cell-cycle arrest (Figure 5C), and apoptosis (Figure 5D), which are reminiscent to the effects of Prmt1 knockdown in MLL-GAS7 and MOZ-TIF2 transformed cells (Figures S1C–S1E). Consistently, Kdm4c knockdown in MLL fusions and MOZ-TIF2 transformed cells resulted in the downregulation of *Hoxa9* (Figure S5B) and the increased level of H3K9me3 repressive marks (Figure S5C). To further eliminate the possibility of any off-target effect of Kdm4c shRNA attributed to the observed transformation phenotype, we co-expressed shRNA-resistant human KDM4C with Kdm4c shRNA in MLL-AF9, MLL-GAS7, and MOZ-TIF2 leukemic cells. As a result, re-expression of KDM4C was able to rescue the transformation defects associated Kdm4c shRNAs (Figure S5D). To investigate whether Kdm4c is also required for maintenance of leukemia in vivo, we transduced MLL fusions and MOZ-TIF2 leukemia cells harvested from primary leukemic mice with a scramble control or the Kdm4c shRNA prior to their transplantation into syngeneic mice for disease development. 72 hours after transplantation, percentages of engraftment were assessed and showed no significant difference between the control and Kdm4c knockdown cells, indicating that Kdm4c knockdown has rather limited impact on homing of the leukemic cells (Figure 5E). In contrast to mice transplanted with control transduced MLL-GAS7 leukemic cells that all succumbed to leukemia within 6 weeks, Kdm4c knockdown in leukemic cells abolished their oncogenic activity, and all mice remained healthy even after 14 weeks of observation (Figures 5F and S5E–S5G). Similarly, inhibition of Kdm4c expression significantly delayed disease latency of MOZ-TIF2-induced leukemia (log-rank test $p < 0.0001$, Figures 5F and S5E–S5G). More importantly, we also observed drastic inhibition of the leukemogenic potentials of Prmt1-independent MLL-AF9 fusion, leading to a significant improvement of the disease-free survival and reduced penetrance (log-rank test $p < 0.0001$, Figures 5F and S5E–S5G). Similar to Prmt1 in vivo knockdown data, the few leukemias from the mice that received Kdm4c-knockdown cells actually re-expressed high levels of Kdm4c and Hoxa9 (Figure S5H), indicating a strong growth disadvantage of Kdm4c knockdown leukemia cells. Together, these results highlight an essential function of Kdm4c in leukemias driven by MLL fusions and MOZ-TIF2.

To further investigate the potential therapeutic window of targeting Kdm4c in the hematopoietic system, we assessed the impact of Kdm4c suppression in normal hematopoiesis. Kdm4c knockdown in c-kit^{+} HSPCs did not lead to any significant reduction of colony-forming ability (Figure 5G) or any change in their ability to differentiate into different lineages (Figure 5H) in vitro. To assess the effect of Kdm4c suppression on in vivo hematopoietic development, we transplanted Kdm4c knockdown HSPCs into sublethally irradiated syngeneic mice for in vivo repopulation assay. Consistent with in vitro data, Kdm4c knockdown cells competently reconstituted the hematopoietic systems (Figure 5I) and gave rise to multiple hematopoietic lineages (including myeloid, B-lymphoid, and T-lymphoid) in a fashion indistinguishable to that in controls 6 weeks after the transplantation (Figure 5J). These results are in line with the dispensable embryonic function of Kdm4c for development into phenotypically normal Kdm4c knockout mouse (Pedersen et al., 2014), suggesting a potential therapeutic window for targeting Kdm4c for leukemia suppression.

Pharmacological Inhibition of Kdm4c Suppresses Leukemia Development in Both Syngeneic Mouse Model and Human AML Xenograft Model

To further demonstrate the therapeutic potentials of targeting KDM4C in AML, we tested the leukemia inhibitory activity of a newly developed KDM4C inhibitor, SD70 (Jin et al., 2014). Using mouse primary cells transformed by MLL fusions and MOZ-TIF2 as the model systems, SD70 was able to significantly suppress their cell growth (Figure 6A), and induced apoptosis (Figure 6B), differentiation (Figure 6C), and cell-cycle arrest (Figure 6D), which are consistent with the effects of Kdm4c knockdown in these cells (Figures 5A–5D). To further access the in vivo efficacy of SD70 treatment, we transplanted MLL-AF9 leukemia cells carrying a luciferase reporter into irradiated syngeneic mice for in vivo treatment with either vehicle control or SD70. By in vivo imaging 6 weeks after the transplant, we detected significant leukemic burdens in the vehicle-treated cohort (Figure 6E). In contrast, SD70 drastically suppressed leukemic burdens (Figure 6E) and, more importantly, significantly extended the disease latency (Figure 6F). Consistent with Kdm4c knockdown data, SD70 was able to suppress H3K9me3 activity in vivo (Figure 6G) and inhibited the expression of MLL downstream target genes (Figure S6A).

To determine whether SD70 can also be effective in human MLL leukemia, we treated various human leukemia cell lines

(D) Venn diagram showing the commonly downregulated genes on MLL-AF9 and MLL-GAS7 after Kdm4c knockdown (left) as well as commonly downregulated pathways identified by GSEA (right).
(E) Embryonic stem cell signature and Myc pathway were downregulated after the loss of Prmt1 or Kdm4c in both MOZ-TIF2 (left) and MLL-GAS7 (right).
(F) Embryonic stem cell signature and Myc pathway were downregulated in both MLL fusions after Kdm4c knockdown.
(G) qRT-PCR analysis of *Myc* and *Bcat1* expression after Kdm4c knockdown.
(H) ChIP analysis showing the effect of Kdm4c knockdown on H3K9me3 mark in *Myc* loci of MLL-AF9 leukemia cells.
All data shown are mean and SD (n = 3) unless otherwise specified. See also Figure S4.

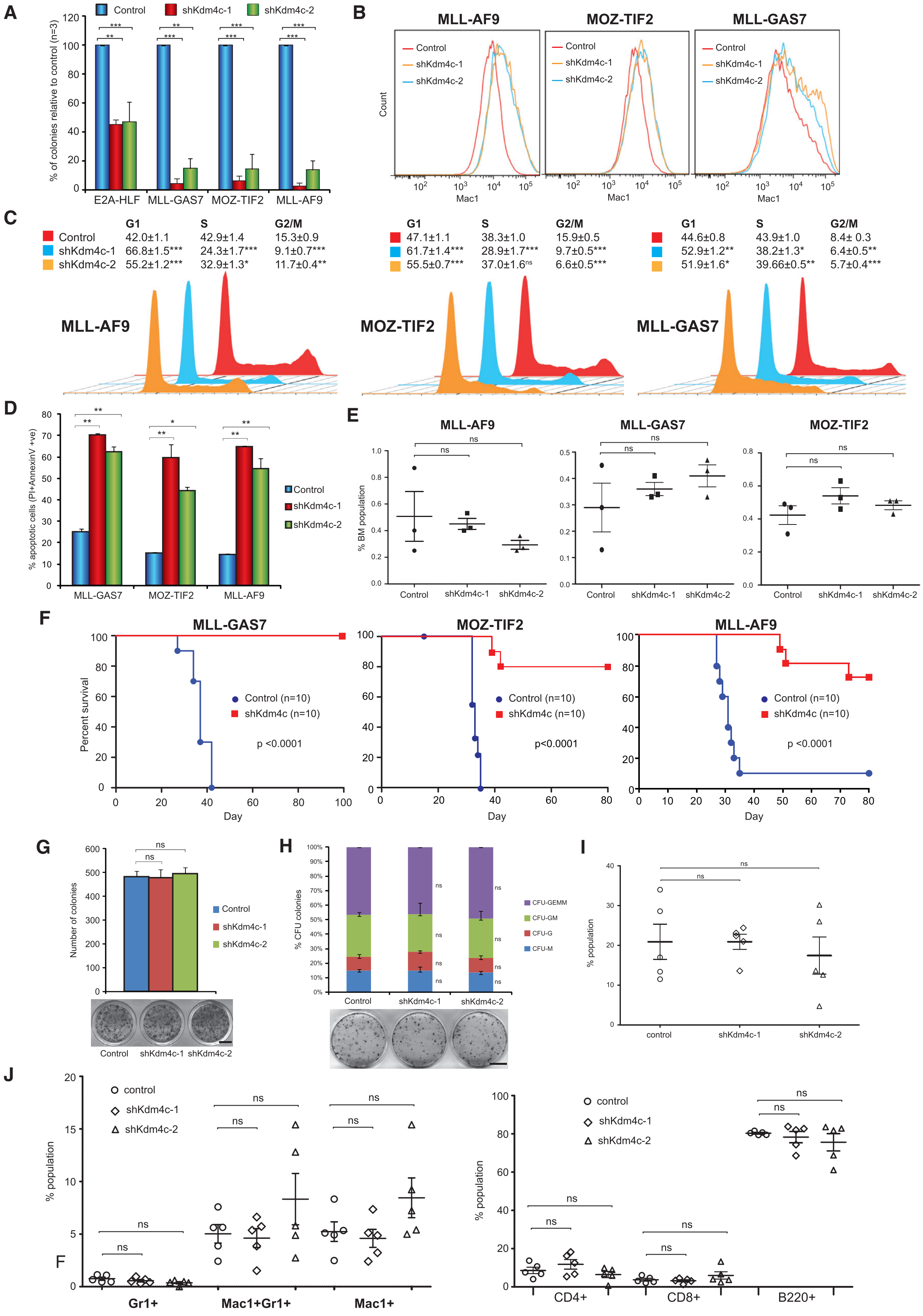

(legend on next page)

carrying different genetic mutations with SD70. As a result, we observed a specific and preferential suppression on human leukemia cell lines carrying MLL fusions (such as SEM and THP1) over the non-MLL leukemia cell lines (Figure 6H). Consistent with the data on mouse primary transformed cells, SD70 induced apoptosis (Figure 6I), differentiation (Figure S6B), and cell-cycle arrest (Figure S6C) accompanied with an increased level of H3K9me3 mark (Figure S6D) in MLL leukemia cell lines, suggesting a similar requirement of KDM4C activity in both human and mouse MLL leukemias.

To further demonstrate the utility of KDM4C inhibitor on the most relevant preclinical setting, we deployed primary AML cells from patients carrying MLL fusions for both in vitro and in vivo drug treatment studies. As a result, we observed that MLL primary leukemia cells (i.e., MLL1-3) were highly sensitive to SD70 (Figures 6J and 6K). A low dose of SD70 (500 nM) efficiently suppressed proliferation (Figure 6J) and induced differentiation (Figure 6K) of primary AML cells carrying MLL fusions (i.e., MLL1-3) but not the control primary AML primary cells without the translocations (non-MLL1-4). To further assess the effects of SD70 on leukemia cell growth and disease development in vivo, we labeled the primary AML cells carrying MLL fusion (MLL3) with a luciferase reporter prior to their transplantation into NSG mice for either the vehicle or SD70 in vivo treatment. Seven weeks after transplantation, in vivo bioluminescence imaging revealed a rapid leukemic growth and disease onset in the control cohort (Figure 6L). In contrast, the SD70-treated cohort had much lower tumor burdens (Figure 6L). More importantly, while the entire control cohort succumbed to leukemia within 59 days (Figures 6M, S6E, and S6F), the SD70-treated group did not show any sign of the disease and all mice survived throughout the observation period (Figures 6M, S6E, and S6F). These results could also be faithfully reproduced using an independent KDM4C shRNA approach on the human MLL3 primary leukemia cells (Figure S6G), where the control cohort with scramble shRNA succumbed to leukemia with a short latency whereas the entire KDM4C knockdown group survived throughout the 90-day observation period (Figure S6G). Together, these results provide the molecular and preclinical evidence for the potential clinical utility of SD70 in MLL leukemia.

DISCUSSION

Elucidation of the mechanisms that orchestrate epigenetic reprogramming by oncogenic transcription factors is critical for understanding the molecular biology of the disease and designing effective therapeutic strategies (Cheung and So, 2011). In this study, we describe the co-recruitment of Prmt1 and Kdm4c by MLL-GAS7 and MOZ-TIF2, which exemplifies the dynamic interplay and cooperation between histone code writers and erasers for execution of specific transcriptional programs mediated by oncogenic transcription factors in acute leukemia (Figure 7). Histone methylation and demethylation as a key component of histone code is on constant flux, and perturbation of this dynamic event on chromatin can shift the equilibrium to alter transcription outcomes. While the collaboration between KMTs and KDMs (e.g., between MLL and JMJD3) (Agger et al., 2007) has been previously documented to facilitate the switch between different transcriptional states by reinforcing specific histone methylation codes on lysine residues (Cheung and So, 2011; Cloos et al., 2008), our study suggests that similar mechanisms also operate between histone arginine and lysine methylations. H4R3me2as encodes an activation mark that allows recruitment of histone acetyltransferases such as CBP and p300 to open up the chromatin structure for gene expression (An et al., 2004; Cheung et al., 2007). To facilitate such a modification, the lysine residues subjected to acetylation should be free from methyl groups. Consistently, recruitment of KDM4C mediates the removal of H3K9me3 repressive mark, allowing the replacement with the activating acetylation mark, suggesting that a coordinated functional recruitment of multiple distinctive epigenetic modifying enzymes is required for establishment of oncogenic transcriptional programs mediated by chimeric transcription factors in cancer development.

As proof of principle, we provide the long-sought-after in vivo preclinical data showing that inhibition of Prmt1 activity by shRNA or chemical inhibitor approaches can significantly suppress oncogenic transformation mediated by various AML fusions, and extend the latency of established disease in the transplanted animals. In recent years, PRMT1 has been implicated in AML1-ETO leukemia (Shia et al., 2012) and some solid tumors (Mathioudaki et al., 2011; Yoshimatsu et al., 2011); however, in vivo transformation data are still required to firmly establish a functional link in the actual disease pathogenesis. The current establishment of an in vivo preclinical model provides a strong rationale and platform to evaluate and develop more specific Prmt1 inhibitors in the future for targeted cancer therapy. Compared with BCR-ABL in CML and PML-RARα in APL, we have an extremely limited knowledge about the molecular functions of PMTs in normal and cancer development

Figure 5. Suppression of Kdm4c Inhibits Hematopoietic Transformation and Leukemogenesis

(A) RTTA showing the effect of two different Kdm4c shRNAs on leukemic transformation.

(B) FACS analysis showing the effect of Kdm4c knockdown on Mac1 expression.

(C) Cell-cycle analysis after Kdm4c knockdown.

(D) Apoptosis analysis after Kdm4c knockdown.

(E) Murine leukemia cells were transduced with the control or shKdm4c lentivirus and transplanted into syngeneic mice. Bone marrows were harvested after 72 hr and the percentage of donor cells was determined by FACS.

(F) Kaplan-Meier survival analysis on the effect of Kdm4c knockdown (shKdm4c#1) on leukemogenesis (log-rank test $p < 0.0001$). Median disease latency: control MLL-AF9, 31 days; MLL-GAS7, 37 days; MOZ-TIF2, 33 days; shKdm4c, undefined.

(G) Effect of Kdm4c knockdown on the colony-forming ability of HSPC. Scale bar represents 0.5 cm.

(H) CFU assay showing the effect of Kdm4c knockdown on the types and number of myeloid colonies derived from HSPC. Scale bar represents 0.5 cm.

(I) FACS analysis of HSPC engraftment using CD45.1 marker to detect the donor cell populations in peripheral blood 6 weeks after transplantation (n = 5).

(J) FACS analysis of the effect of Kdm4c knockdown on both myeloid (left) and lymphoid populations (right) in peripheral blood (n = 5).

All data shown are mean and SD (n = 3) unless otherwise specified. See also Figure S5.

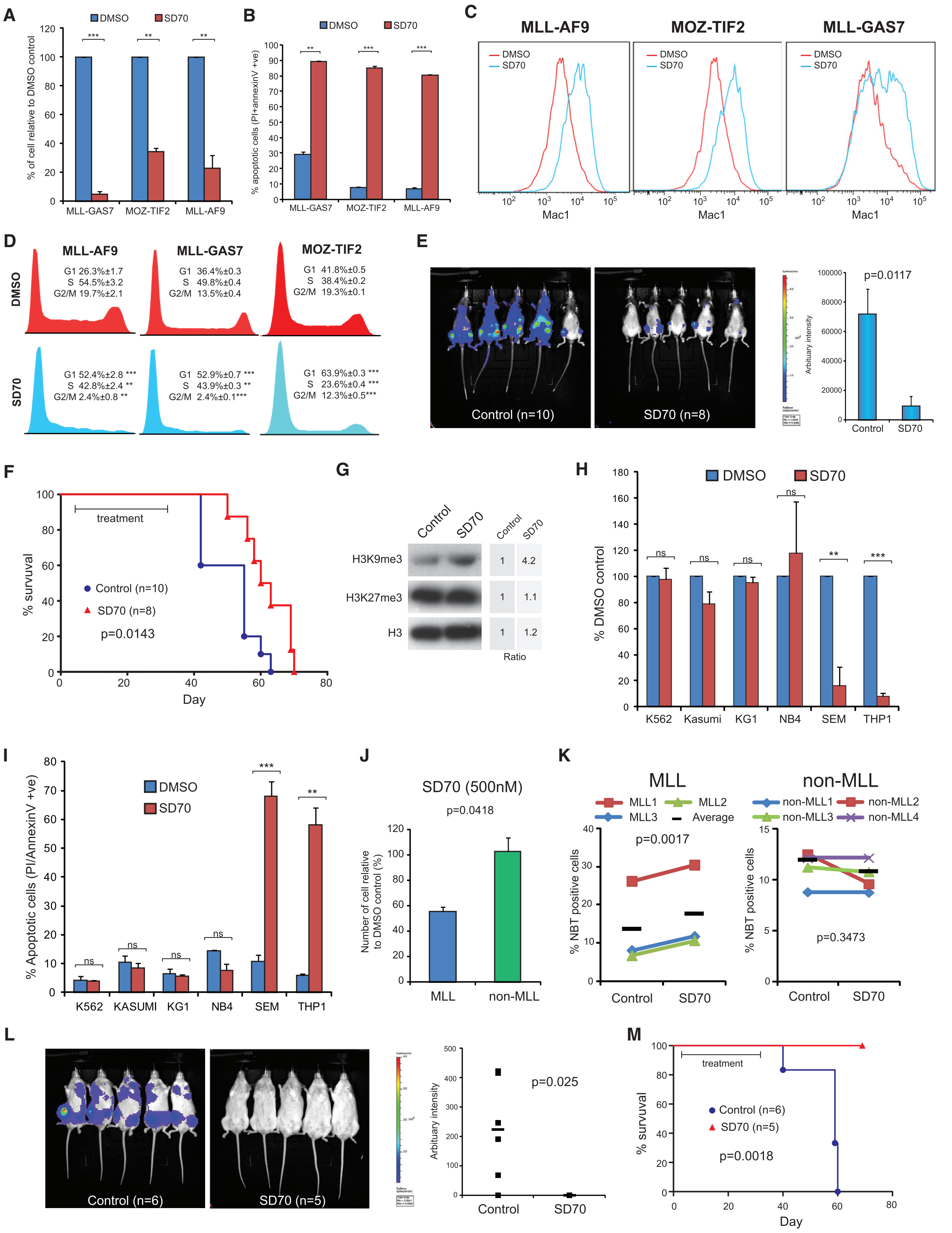

(legend on next page)

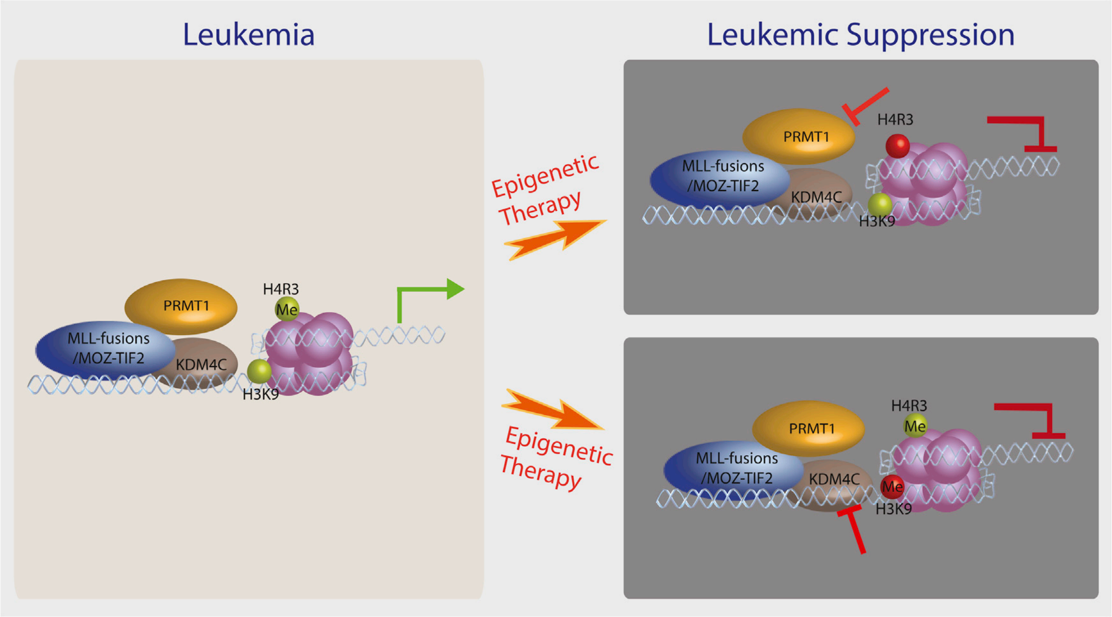

Figure 7. Schematic Diagram Summarizes Aberrant Epigenetic Networks and Therapeutic Potentials of Targeting PRMT1 and KDM4C in AML

Left panel indicates the aberrant recruitment of PRMT1 and KDM4C by MLL fusions and MOZ-TIF2 to drive oncogenic transcriptional programs. Panels on the right depict the potential targeting of epigenetic modifying enzymes for leukemia suppression. MLL fusions in the diagram refer to MLL-GAS7 and MLL-EEN, although KDM4C inhibition can also be effective for other MLL fusions including MLL-AF9.

despite a preliminary indication of clinical efficacy of the KMT inhibitor against DOT1L in the phase I trial. The lessons from imatinib on CML (Balabanov et al., 2014) and ATRA on APL (Fung and So, 2013) indicate that the development of drug-resistant clones after achieving an initial clinical remission by highly effective and specific inhibitors against a particular molecule will be a major issue for most of the targeted therapies. Understanding the mechanisms of action is essential, and has been instrumental in designing more effective therapeutic strategies to minimize and overcome relapses (Cortes et al., 2012; Lo-Coco et al., 2013). The discovery of the functional crosstalk between PRMT1 and KDM4C in the establishment of an oncogenic transcriptional program in leukemia provides important insights into the molecular functions and underlying mechanisms of these critical PMTs and KMDs in oncogenesis.

The dynamics of H3K9 methylation is regulated by the intricate equilibrium of lysine methyltransferases and demethylases. Using in vitro cell line models, KDM4C has been implicated in different cancers including squamous cell carcinoma (Cloos et al., 2006), B-cell lymphoma (Rui et al., 2010), and prostate (Wissmann et al., 2007), and breast (Liu et al., 2009) cancers. Loss of Suv39h resulted in a reduction of H3K9me3 mark and accelerated tumor development (Braig et al., 2005; Peters et al., 2001), suggesting a tumor-suppressor function associated with H3K9me3 in critical but unknown loci. Consistent with this model, recruitment of KDM4C by chimeric transcription factors

Figure 6. SD70 Inhibits Leukemogenesis In Vitro and In Vivo

(A) Murine leukemia cells were treated with SD70 or DMSO for 3 days with cell viability determined by trypan blue exclusion assay.
(B) Apoptosis analysis on SD70- or DMSO-treated murine leukemia cells.
(C) FACS analysis of myeloid marker Mac1 in murine leukemia cells after SD70 treatment.
(D) Cell-cycle analysis after SD70 treatment.
(E) MLL-AF9-luciferase leukemia cells were transplanted into syngeneic mice and subjected to either vehicle control or SD70 treatment. Bioluminescence imaging was performed at 39 days after transplantation.
(F) Kaplan-Meier survival analysis on the effect of SD70 treatment on MLL-AF9 mediated leukemogenesis (log-rank test $p = 0.0143$). Median disease latency: control, 55 days; SD70, 61.5 days.
(G) Western blotting analysis of H3K9me3 mark in murine MLL-AF9 leukemia cells after SD70 in vivo treatment. Intensity ratio was determined by densitometry.
(H) Cell viability of human leukemia cell lines were determined 3 days after SD70 or DMSO treatment.
(I) Apoptosis analysis of SD70-treated human leukemia cell lines.
(J) Cell viability of primary AML patient samples after SD70 treatment (MLL, $n = 3$; non-MLL, $n = 4$).
(K) NBT reduction assay to determine myeloid differentiation of MLL and non-MLL patient samples. The black bars show the mean.
(L) Primary human MLL leukemia (MLL3) was tagged with luciferase reporter and then transplanted into NSG mice. Bioluminescence imaging of control ($n = 6$) and SD70-treated ($n = 5$) cohorts were performed on day 44 after transplantation. Bars show the mean bioluminescence intensity.
(M) Kaplan-Meier survival analysis of vehicle or SD70-treated cohort transplanted with primary human MLL3 leukemia cells (log-rank test $p = 0.0018$). Median disease latency: control, 59 days; SD70, undefined.
All data shown are mean and SD ($n = 3$) unless otherwise specified. See also Figure S6.

can counteract and remove the repressive H3K9 trimethylation marks on their target gene loci such as *Hoxa9*, which are critical for self-renewal and oncogenic transformation. This is supported by the concomitant increase in H3K9me3 mark and suppression of *Hoxa9* expression upon Kdm4c knockdown. Recently, H3K9me2/1 demethylase PHF8 has been shown to govern ATRA treatment response in APL, and its activation helps to overcome treatment resistance (Arteaga et al., 2013). Our current studies provide the key in vivo experimental evidence demonstrating the requirement of KDM4C for cancer development and its functional crosstalk with PRMT1 in the establishment of histone codes for transcriptional deregulation in AML. Intriguingly, suppression of either of the epigenetic regulators compromises transcriptional programs and cellular transformation by MOZ-TIF2 or MLL-GAS7 fusions, indicating their critical and non-overlapping functions that are indispensable for the oncogenic transformation (Figure 7). This is also consistent with the finding that KDM4C is required for PRMT1-independent MLL leukemia. Importantly, transcriptional or pharmacological inhibition of KDM4C by molecular or small-molecule inhibitor approaches could significantly lower leukemic burdens and extend the disease latency, particularly in an MLL primary human AML cell xenograft in vivo model. Together, these findings provide strong experimental and preclinical in vivo evidence demonstrating an efficient MLL leukemia suppression by pharmacological inhibition of KDM4C, laying the foundation for future clinical application of KDM4C inhibitors in human cancer treatments.

Advancement in our knowledge of onco-epigenomics, together with the dissection of the dynamic interplay of chromatin modification and remodeling mediated by leukemic transcription factors, could pave the way to revolutionize our therapeutic options. Cracking the lethal histone code created by leukemic fusions and a strategy of rational therapeutic design targeting specific epigenetic modifying enzymes required for the oncogenic transcription factors hold the promise of eradicating this devastating disease.

EXPERIMENTAL PROCEDURES

In Vitro and In Vivo Transformation Studies

RTTA was performed as previously described (Zeisig and So, 2009) and is detailed in Supplemental Experimental Procedures. For Kdm4c knockdown rescue experiments, cells were co-transduced with Kdm4c shRNA and shRNA-resistant human KDM4C lentiviruses, and co-selected with puromycin and blasticidin. To generate full-blown murine leukemia cells, we injected 10^6 immortalized cells via the tail vein into sublethally irradiated syngeneic C57BL/6 mice. Mice were injected with 10^5 murine leukemia cells to study the effect Prmt1 and Kdm4c knockdown on leukemogenesis. Leukemia cells were transduced with either control or shPrmt1 retrovirus, and GFP sorted before transplantation. Kdm4c knockdown cells were selected with antibiotic prior to transplantation. Prmt1 knockout induced by tamoxifen were confirmed by PCR genotyping prior to transplantation. Mice were monitored for development of leukemia by fluorescence-activated cell sorting (FACS) analysis with tissues processed for histological analysis. Primary human MLL3 leukemia cells were transduced with control or shKDM4C lentivirus, and antibiotic selected and transplanted (10^5) by intrafemoral injection into sublethally irradiated *NOD/SCID/IL2Rg*$^{-/-}$ (NSG) mice. For bioluminescence imaging and quantification of leukemia burden, murine leukemias were established using HSPC isolated from Ubc-luciferase reporter mice (Becker et al., 2006), whereas human leukemia was tagged with a lentiviral luciferase reporter. Transplanted mice were injected with 150 mg/kg of D-luciferin intraperitoneally and bioluminescence image acquired using IVIS Lumina II (Caliper; Perkin Elmer) with software Living Image Version 4.3.1 according to the manufacturer's instructions. All the animal works were performed according to the guidelines and regulations of the Animal (Scientific Procedures) Act 1986, and approved by the KCL local ethics committee.

AMI-408 Drug Treatment

For AMI-408 drug treatment in vitro, 10^4 cells were plated in methylcellulose with 200 mM AMI-408 or DMSO control and cultured for 5–7 days. To study the effect of AMI-408 on leukemogenesis of MLL-GAS7 in vivo, we pretreated leukemia cells in vitro with the drug for 24 hr prior to injection. 10^5 MLL-GAS7 leukemia cells were then transplanted into C57BL/6 mice via tail vein and injected intraperitoneally with 20 mg/kg of AMI-408 or carrier control every other day for 2 weeks. To study the effect of AMI-408 on MOZ-TIF2 leukemogenesis in vivo, we transplanted 10^5 leukemia cells into syngeneic C57BL/6 mice via tail vein injection without pretreatment. Mice were subjected to a dosage of 10 mg/kg of AMI-408 in PEG300/D5W for 4 weeks with five consecutive injections per week. Bioluminescence imaging was performed every week.

SD70 Drug Treatment

Leukemia cells were seeded at 5×10^4/ml and incubated with SD70 (Xcess Biosciences) at a concentration of 0.8 μM for human cell lines, 2 μM for murine, and 0.5 μM for primary human samples for 48–72 hr. To study the effect of SD70 on leukemogenesis in vivo, we transplanted 10^5 murine MLL-AF9-luciferase leukemia cells 3 days prior to treatment. Human primary leukemia MLL3 was transplanted by intrafemoral injection into sublethally irradiated NSG mice 3 days before drug treatment. SD70 preparation and drug dosage used for in vivo animal experiments were performed as described by Jin et al. (2014). SD70 was administered intraperitoneally at 10 mg/kg in PEG300/D5W for 4 weeks with five consecutive injections in the first week and every alternative day for the next 3 weeks. Sick mice were euthanized and processed for FACS and histological analysis. SD70-treated mice were injected with 50 mg/kg of SD70 intraperitoneally, and bone marrow was harvested 5 hr later for western blot analysis.

Statistical Analysis

All the experiments were analyzed using two-way Student's t test. For the comparison of different specimens the unpaired t test was used. For the comparison of different treatments (e.g., drug, gene knockdown/knockout) within the same specimen, the paired t test was used. The log-rank test was used to compare survival curves. p Values of less than 0.05 were considered statistically significant. In the figures, asterisks indicate $*p < 0.05$, $**p < 0.01$, and $***p < 0.001$. For the RNA-seq analysis, differentially expressed genes with $p < 0.05$ were used to generate heatmaps. The statistical significance of overlap between the gene expression patterns of two conditions was calculated using a hypergeometric test (Marioni et al., 2008).

Additional experimental procedures including description of plasmids, cell lines, conditions for qRT-PCR, GST pull-down assay, immunoprecipitation, chromatin immunoprecipitation, NTB reduction assay, flow cytometry, generation of Prmt1 conditional knockout mouse, RNA-seq, and RNA analysis are reported in Supplemental Experimental Procedures.

ACCESSION NUMBERS

RNA-sequencing data can be accessed via ArrayExpress: E-MTAB-3322.

SUPPLEMENTAL INFORMATION

Supplemental Information includes Supplemental Experimental Procedures, six figures, and three tables and can be found with this article online at http://dx.doi.org/10.1016/j.ccell.2015.12.007.

ACKNOWLEDGMENTS

We would like to dedicate this paper to the memory of Prof. Li Chong Chan for his important contributions to the foundation of this work, which was started 20 years ago. Thanks are given to Jackie Chen and Amanda Wilson for assistance

on various experimental procedures; Sam Tung, Magdalena Zarowiecki, and members of Lenhard's laboratory for advice on bioinformatics analysis; Jesus Gil, Hinrich Gronemeyer, Peter Parker, Joyce Taylor-Papdadimitriou, and Joy Burchell for critical advice on the manuscript; Erica Tse for graphic illustration; and KCL National Institute for Health Research Biomedical Research Center (NIHR BRC) for sequencing service. This research was funded by Bloodwise with additional support from Cancer Research UK (CRUK) to C.W.E.S.

Received: February 13, 2015
Revised: July 31, 2015
Accepted: December 15, 2015
Published: January 11, 2016

REFERENCES

Abdel-Wahab, O., and Levine, R.L. (2013). Mutations in epigenetic modifiers in the pathogenesis and therapy of acute myeloid leukemia. Blood *121*, 3563–3572.

Agger, K., Cloos, P.A., Christensen, J., Pasini, D., Rose, S., Rappsilber, J., Issaeva, I., Canaani, E., Salcini, A.E., and Helin, K. (2007). UTX and JMJD3 are histone H3K27 demethylases involved in HOX gene regulation and development. Nature *449*, 731–734.

An, W., Kim, J., and Roeder, R.G. (2004). Ordered cooperative functions of PRMT1, p300, and CARM1 in transcriptional activation by p53. Cell *117*, 735–748.

Arteaga, M.F., Mikesch, J.H., Qiu, J., Christensen, J., Helin, K., Kogan, S.C., Dong, S., and So, C.W. (2013). The histone demethylase PHF8 governs retinoic acid response in acute promyelocytic leukemia. Cancer Cell *23*, 376–389.

Arteaga, M.F., Mikesch, J.H., Fung, T.K., and So, C.W. (2015). Epigenetics in acute promyelocytic leukaemia pathogenesis and treatment response: a TRAnsition to targeted therapies. Br. J. Cancer *112*, 413–418.

Balabanov, S., Braig, M., and Brummendorf, T.H. (2014). Current aspects in resistance against tyrosine kinase inhibitors in chronic myelogenous leukemia. Drug Discov. Today Technol. *11*, 89–99.

Becker, C.M., Wright, R.D., Satchi-Fainaro, R., Funakoshi, T., Folkman, J., Kung, A.L., and D'Amato, R.J. (2006). A novel noninvasive model of endometriosis for monitoring the efficacy of antiangiogenic therapy. Am. J. Pathol. *168*, 2074–2084.

Bonham, K., Hemmers, S., Lim, Y.H., Hill, D.M., Finn, M.G., and Mowen, K.A. (2010). Effects of a novel arginine methyltransferase inhibitor on T-helper cell cytokine production. FEBS J. *277*, 2096–2108.

Braig, M., Lee, S., Loddenkemper, C., Rudolph, C., Peters, A.H., Schlegelberger, B., Stein, H., Dorken, B., Jenuwein, T., and Schmitt, C.A. (2005). Oncogene-induced senescence as an initial barrier in lymphoma development. Nature *436*, 660–665.

Campbell, R.M., and Tummino, P.J. (2014). Cancer epigenetics drug discovery and development: the challenge of hitting the mark. J. Clin. Invest. *124*, 64–69.

Cheung, N., and So, C.W. (2011). Transcriptional and epigenetic networks in haematological malignancy. FEBS Lett. *585*, 2100–2111.

Cheung, N., Chan, L.C., Thompson, A., Cleary, M.L., and So, C.W. (2007). Protein arginine-methyltransferase-dependent oncogenesis. Nat. Cell Biol. *9*, 1208–1215.

Cloos, P.A., Christensen, J., Agger, K., Maiolica, A., Rappsilber, J., Antal, T., Hansen, K.H., and Helin, K. (2006). The putative oncogene GASC1 demethylates tri- and dimethylated lysine 9 on histone H3. Nature *442*, 307–311.

Cloos, P.A., Christensen, J., Agger, K., and Helin, K. (2008). Erasing the methyl mark: histone demethylases at the center of cellular differentiation and disease. Genes Dev. *22*, 1115–1140.

Cortes, J.E., Kantarjian, H., Shah, N.P., Bixby, D., Mauro, M.J., Flinn, I., O'Hare, T., Hu, S., Narasimhan, N.I., Rivera, V.M., et al. (2012). Ponatinib in refractory Philadelphia chromosome-positive leukemias. N. Engl. J. Med. *367*, 2075–2088.

Daigle, S.R., Olhava, E.J., Therkelsen, C.A., Majer, C.R., Sneeringer, C.J., Song, J., Johnston, L.D., Scott, M.P., Smith, J.J., Xiao, Y., et al. (2011). Selective killing of mixed lineage leukemia cells by a potent small-molecule DOT1L inhibitor. Cancer Cell *20*, 53–65.

Dawson, M.A., Prinjha, R.K., Dittmann, A., Giotopoulos, G., Bantscheff, M., Chan, W.I., Robson, S.C., Chung, C.W., Hopf, C., Savitski, M.M., et al. (2011). Inhibition of BET recruitment to chromatin as an effective treatment for MLL-fusion leukaemia. Nature *478*, 529–533.

Esposito, M.T., Zhao, L., Fung, T.K., Rane, J.K., Wilson, A., Martin, N., Gil, J., Leung, A.Y., Ashworth, A., and So, C.W. (2015). Synthetic lethal targeting of oncogenic transcription factors in acute leukemia by PARP inhibitors. Nat. Med. *21*, 1481–1490.

Fung, T.K., and So, C.W. (2013). Overcoming treatment resistance in acute promyelocytic leukemia and beyond. Oncotarget *4*, 1128–1129.

He, J., Nguyen, A.T., and Zhang, Y. (2011). KDM2b/JHDM1b, an H3K36me2-specific demethylase, is required for initiation and maintenance of acute myeloid leukemia. Blood *117*, 3869–3880.

Huntly, B.J., Shigematsu, H., Deguchi, K., Lee, B.H., Mizuno, S., Duclos, N., Rowan, R., Amaral, S., Curley, D., Williams, I.R., et al. (2004). MOZ-TIF2, but not BCR-ABL, confers properties of leukemic stem cells to committed murine hematopoietic progenitors. Cancer Cell *6*, 587–596.

Ingham, R.J., Colwill, K., Howard, C., Dettwiler, S., Lim, C.S.H., Yu, J., Hersi, K., Raaijmakers, J., Gish, G., Mbamalu, G., et al. (2005). WW domains provide a platform for the assembly of multiprotein networks. Mol. Cell Biol. *25*, 7092–7106.

Jin, C., Yang, L., Xie, M., Lin, C., Merkurjev, D., Yang, J.C., Tanasa, B., Oh, S., Zhang, J., Ohgi, K.A., et al. (2014). Chem-seq permits identification of genomic targets of drugs against androgen receptor regulation selected by functional phenotypic screens. Proc. Natl. Acad. Sci. USA *111*, 9235–9240.

Kantarjian, H., Sawyers, C., Hochhaus, A., Guilhot, F., Schiffer, C., Gambacorti-Passerini, C., Niederwieser, D., Resta, D., Capdeville, R., Zoellner, U., et al. (2002). Hematologic and cytogenetic responses to imatinib mesylate in chronic myelogenous leukemia. N. Engl. J. Med. *346*, 645–652.

Katsumoto, T., Aikawa, Y., Iwama, A., Ueda, S., Ichikawa, H., Ochiya, T., and Kitabayashi, I. (2006). MOZ is essential for maintenance of hematopoietic stem cells. Genes Dev. *20*, 1321–1330.

Knutson, S.K., Wigle, T.J., Warholic, N.M., Sneeringer, C.J., Allain, C.J., Klaus, C.R., Sacks, J.D., Raimondi, A., Majer, C.R., Song, J., et al. (2012). A selective inhibitor of EZH2 blocks H3K27 methylation and kills mutant lymphoma cells. Nat. Chem. Biol. *8*, 890–896.

Kouzarides, T. (2007). Chromatin modifications and their function. Cell *128*, 693–705.

Kvinlaug, B.T., Chan, W.I., Bullinger, L., Ramaswami, M., Sears, C., Foster, D., Lazic, S.E., Okabe, R., Benner, A., Lee, B.H., et al. (2011). Common and overlapping oncogenic pathways contribute to the evolution of acute myeloid leukemias. Cancer Res. *71*, 4117–4129.

Liu, G., Bollig-Fischer, A., Kreike, B., van de Vijver, M.J., Abrams, J., Ethier, S.P., and Yang, Z.Q. (2009). Genomic amplification and oncogenic properties of the GASC1 histone demethylase gene in breast cancer. Oncogene *28*, 4491–4500.

Lo-Coco, F., Avvisati, G., Vignetti, M., Thiede, C., Orlando, S.M., Iacobelli, S., Ferrara, F., Fazi, P., Cicconi, L., Di Bona, E., et al. (2013). Retinoic acid and arsenic trioxide for acute promyelocytic leukemia. N. Engl. J. Med. *369*, 111–121.

Look, A.T. (1997). Oncogenic transcription factors in the human acute leukemias. Science *278*, 1059–1064.

Marioni, J.C., Mason, C.E., Mane, S.M., Stephens, M., and Gilad, Y. (2008). RNA-seq: an assessment of technical reproducibility and comparison with gene expression arrays. Genome Res. *18*, 1509–1517.

Mathioudaki, K., Scorilas, A., Ardavanis, A., Lymberi, P., Tsiambas, E., Devetzi, M., Apostolaki, A., and Talieri, M. (2011). Clinical evaluation of PRMT1 gene expression in breast cancer. Tumour Biol. *32*, 575–582.

McCabe, M.T., Ott, H.M., Ganji, G., Korenchuk, S., Thompson, C., Van Aller, G.S., Liu, Y., Graves, A.P., Della Pietra, A., 3rd, Diaz, E., et al. (2012). EZH2 inhibition as a therapeutic strategy for lymphoma with EZH2-activating mutations. Nature *492*, 108–112.

Okada, Y., Feng, Q., Lin, Y., Jiang, Q., Li, Y., Coffield, V.M., Su, L., Xu, G., and Zhang, Y. (2005). hDOT1L links histone methylation to leukemogenesis. Cell *121*, 167–178.

Pedersen, M.T., Agger, K., Laugesen, A., Johansen, J.V., Cloos, P.A., Christensen, J., and Helin, K. (2014). The demethylase JMJD2C localizes to H3K4me3-positive transcription start sites and is dispensable for embryonic development. Mol. Cell Biol. *34*, 1031–1045.

Peters, A.H., O'Carroll, D., Scherthan, H., Mechtler, K., Sauer, S., Schofer, C., Weipoltshammer, K., Pagani, M., Lachner, M., Kohlmaier, A., et al. (2001). Loss of the Suv39h histone methyltransferases impairs mammalian heterochromatin and genome stability. Cell *107*, 323–337.

Rui, L., Emre, N.C., Kruhlak, M.J., Chung, H.J., Steidl, C., Slack, G., Wright, G.W., Lenz, G., Ngo, V.N., Shaffer, A.L., et al. (2010). Cooperative epigenetic modulation by cancer amplicon genes. Cancer Cell *18*, 590–605.

Shia, W.J., Okumura, A.J., Yan, M., Sarkeshik, A., Lo, M.C., Matsuura, S., Komeno, Y., Zhao, X., Nimer, S.D., Yates, J.R., 3rd, and Zhang, D.E. (2012). PRMT1 interacts with AML1-ETO to promote its transcriptional activation and progenitor cell proliferative potential. Blood *119*, 4953–4962.

Shih, A.H., Abdel-Wahab, O., Patel, J.P., and Levine, R.L. (2012). The role of mutations in epigenetic regulators in myeloid malignancies. Nat. Rev. Cancer *12*, 599–612.

So, C.W., Karsunky, H., Passegue, E., Cozzio, A., Weissman, I.L., and Cleary, M.L. (2003a). MLL-GAS7 transforms multipotent hematopoietic progenitors and induces mixed lineage leukemias in mice. Cancer Cell *3*, 161–171.

So, C.W., Lin, M., Ayton, P.M., Chen, E.H., and Cleary, M.L. (2003b). Dimerization contributes to oncogenic activation of MLL chimeras in acute leukemias. Cancer Cell *4*, 99–110.

Somervaille, T.C., Matheny, C.J., Spencer, G.J., Iwasaki, M., Rinn, J.L., Witten, D.M., Chang, H.Y., Shurtleff, S.A., Downing, J.R., and Cleary, M.L. (2009). Hierarchical maintenance of MLL myeloid leukemia stem cells employs a transcriptional program shared with embryonic rather than adult stem cells. Cell Stem Cell *4*, 129–140.

Sroczynska, P., Cruickshank, V.A., Bukowski, J.P., Miyagi, S., Bagger, F.O., Walfridsson, J., Schuster, M.B., Porse, B., and Helin, K. (2014). shRNA screening identifies JMJD1C as being required for leukemia maintenance. Blood *123*, 1870–1882.

Wang, Z.Y., and Chen, Z. (2008). Acute promyelocytic leukemia: from highly fatal to highly curable. Blood *111*, 2505–2515.

Wissmann, M., Yin, N., Muller, J.M., Greschik, H., Fodor, B.D., Jenuwein, T., Vogler, C., Schneider, R., Gunther, T., Buettner, R., et al. (2007). Cooperative demethylation by JMJD2C and LSD1 promotes androgen receptor-dependent gene expression. Nat. Cell Biol. *9*, 347–353.

Wong, S.H., Goode, D.L., Iwasaki, M., Wei, M.C., Kuo, H.P., Zhu, L., Schneidawind, D., Duque-Afonso, J., Weng, Z., and Cleary, M.L. (2015). The H3K4-methyl epigenome regulates leukemia stem cell oncogenic potential. Cancer Cell *28*, 198–209.

Yoshimatsu, M., Toyokawa, G., Hayami, S., Unoki, M., Tsunoda, T., Field, H.I., Kelly, J.D., Neal, D.E., Maehara, Y., Ponder, B.A., et al. (2011). Dysregulation of PRMT1 and PRMT6, Type I arginine methyltransferases, is involved in various types of human cancers. Int. J. Cancer *128*, 562–573.

Zeisig, B.B., and So, C.W. (2009). Retroviral/lentiviral transduction and transformation assay. Methods Mol. Biol. *538*, 207–229.

Zeisig, B.B., Kulasekararaj, A.G., Mufti, G.J., and So, C.W. (2012). Acute myeloid leukemia: snapshot. Cancer Cell *22*, 698.

Zuber, J., Shi, J., Wang, E., Rappaport, A.R., Herrmann, H., Sison, E.A., Magoon, D., Qi, J., Blatt, K., Wunderlich, M., et al. (2011). RNAi screen identifies Brd4 as a therapeutic target in acute myeloid leukaemia. Nature *478*, 524–528.

Cancer Cell, Volume *29*

Supplemental Information

Targeting Aberrant Epigenetic Networks Mediated by PRMT1 and KDM4C in Acute Myeloid Leukemia

Ngai Cheung, Tsz Kan Fung, Bernd B. Zeisig, Katie Holmes, Jayant K. Rane, Kerri A. Mowen, Michael G. Finn, Boris Lenhard, Li Chong Chan, and Chi Wai Eric So

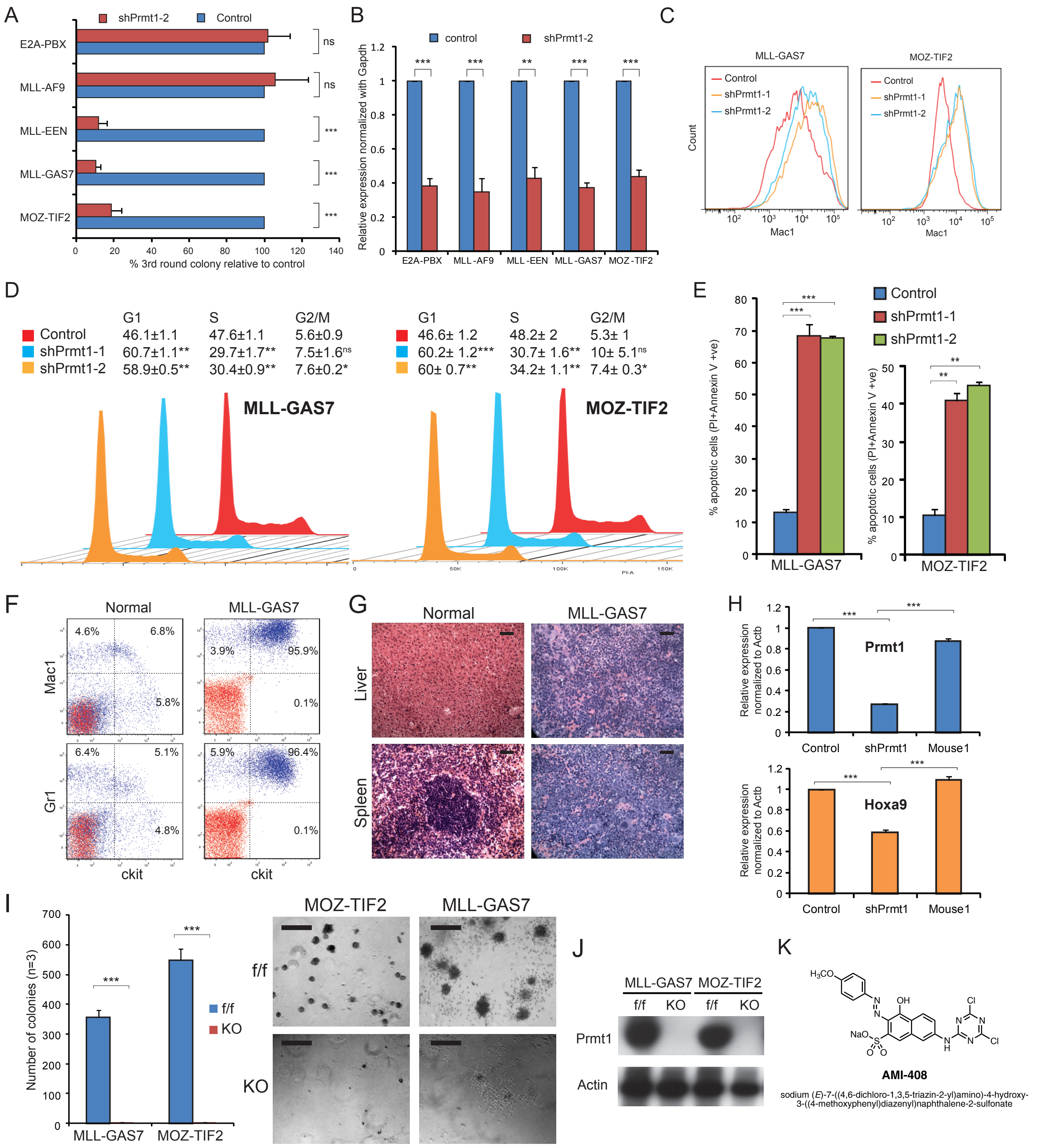

Figure S1, related to Figure 1. Effect of Prmt1 knockdown and AMI-408 treatment on leukemogenesis

A. Prmt1 knockdown using shPrmt1-2 specifically suppresses MLL-EEN, MLL-GAS7 and MOZ-TIF2 colony formation in replating assay. Shown are mean and SD (n=3).

B. RT-qPCR validation of Prmt1 knockdown by shPrmt1-2 in leukemic cells as indicated. Shown are mean and SD (n=3).

C. Prmt1 knockdown in MLL-GAS7 and MOZ-TIF2 leukemia cells induces the up-regulation of myeloid surface marker Mac1 expression as revealed by FACS analysis.

D. Cell cycle analysis revealed an increased in G1 and decreased S phase population in both MLL-GAS7 and MOZ-TIF2 leukemia cells after Prmt1 knockdown. Shown are mean and SD (n=3).

E. Prmt1 knockdown in MLL-GAS7 and MOZ-TIF2 leukemia cells with two independent Prmt1 shRNA results in increased apoptotic cell death as revealed by Annexin V-PI staining. Shown are mean and SD (n=3).

F. FACS analysis of the bone marrow of normal, MLL-GAS7 leukemic mice showing the expression of c-kit and both myeloid markers Mac1 and Gr1 in leukemic blasts.

G. Histological analysis of H&E tissue sections showing the infiltration of leukemic blasts into liver and spleen of leukemic mice as indicated. The normal spleen and liver are also shown as references. Scale bar indicate 25 μm.

H. Some of the mice transplanted with Prmt1 knockdown leukemia cells eventually developed acute myeloid leukemia with a longer latency and lower disease penetrance. Quantitative RT-PCR analysis in those leukemia cells (Mouse 1) revealed a comparable expression level of *Prmt1* and *Hoxa9* mRNA to control that indicative of the loss of Prmt1 down-regulation in those leukemia cells.

I. Prmt1 knockout (KO) in MLL-GAS7 and MOZ-TIF2 suppress colony formation in methylcellulose replating assay. “f/f” was served as control. Shown are mean and SD (n=3). Scale bar indicate 5 mm.

J. Prmt1 KO in both MLL-GAS7 and MOZ-TIF2 leukemia cells was confirmed by Western blot with anti-Prmt1 and actin antibodies.

K. Structure of compound AMI-408.

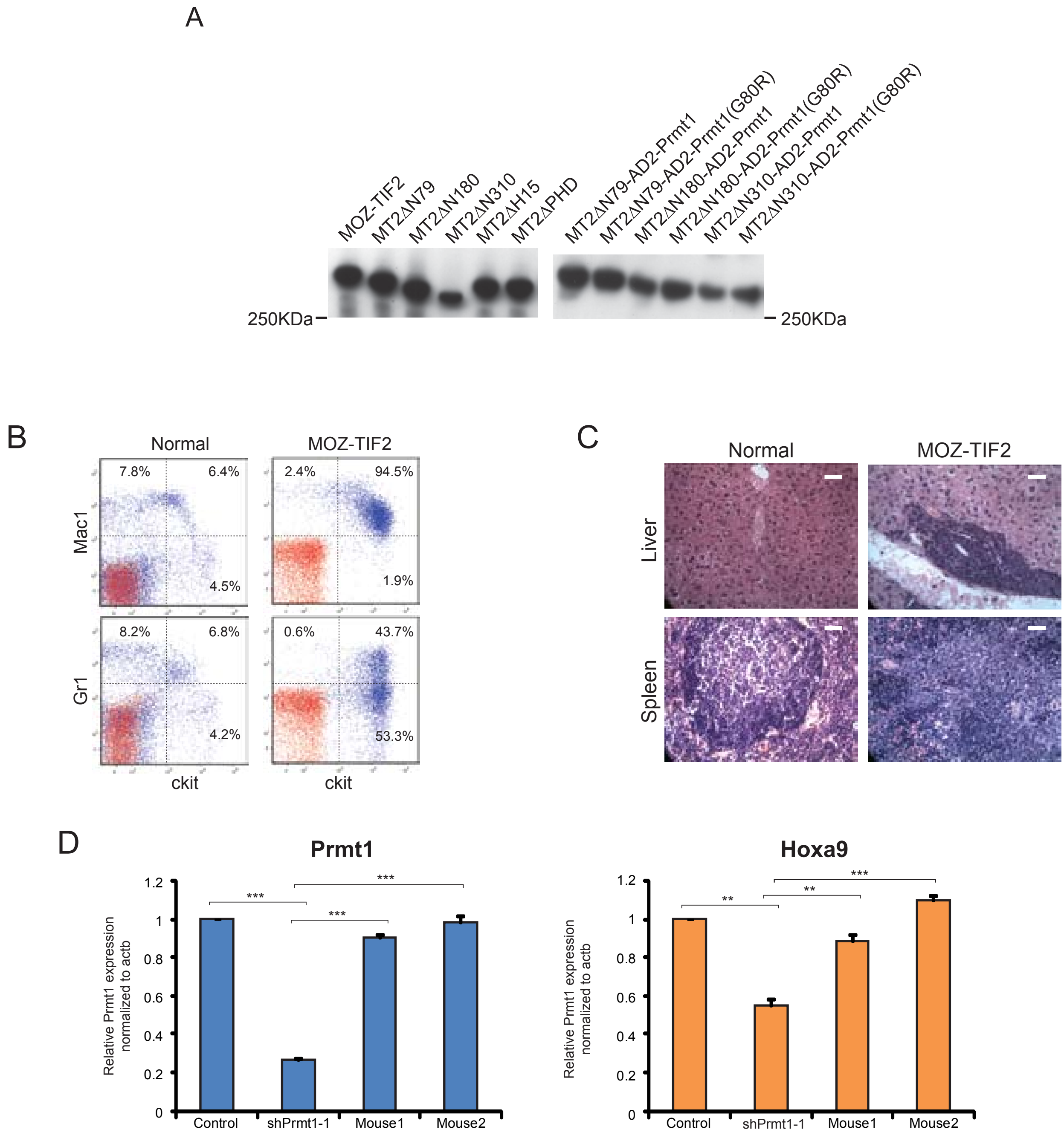

Figure S2, related to figure 2. Effect of Prmt1 knockdown on MOZ-TIF2 leukemia cells in vitro & in vivo

A. Western blot showed the expression of FLAG-tagged MOZ-TIF2, deletion mutants and its Prmt1 rescue fusions in transduced cells.

B. FACS analysis of the bone marrow of normal and MOZ-TIF2 leukemia mice showing the expression of c-kit and both myeloid markers Mac1 and Gr1 in leukemic blasts.

C. Examination of tissue sections show the infiltration of leukemic blasts into liver and spleen of leukemic mice. Scale bar indicate 25 μM.

D. Some of the mice transplanted with Prmt1 knockdown leukemia cells developed acute myeloid leukemia with a longer latency and lower disease penetrance. Quantitative RT-PCR analysis of those leukemia cells (Mouse 1 and 2) revealed a comparable expression level of *Prmt1* (left panel) and *Hoxa9* (right panel) mRNA to control that indicative of the loss of Prmt1 down-regulation in those leukemia cells.

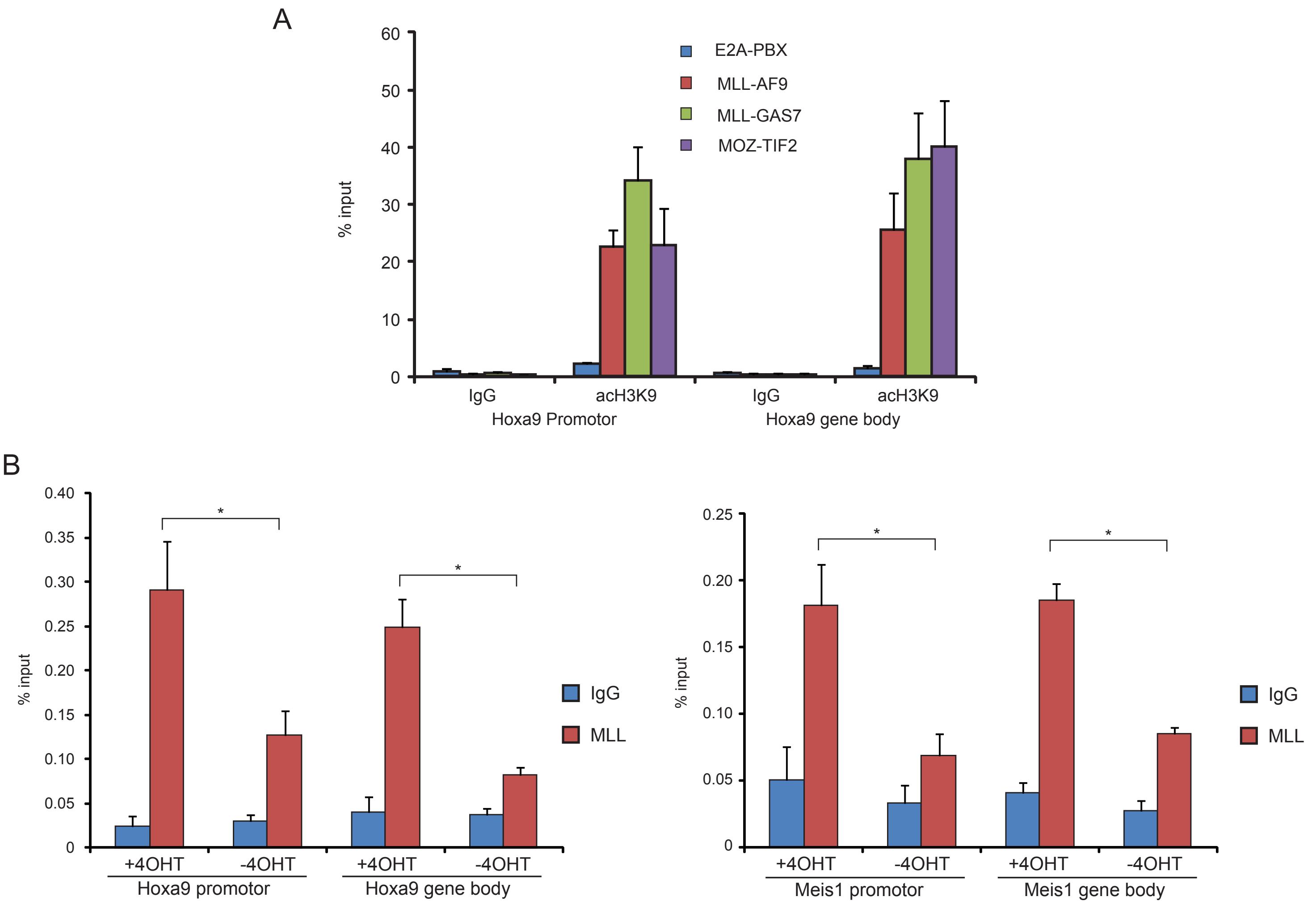

Figure S3, related to Figure 3. ChIP analysis of H3K9ac on leukemic cells and MLL binding at Hoxa9 loci in MLL-AF9-ER cells

A. Increased H3K9 acetylation in *Hoxa9* loci were detected in MLL-AF9, MLL-GAS7 and MOZ-TIF2 leukemic cells but not in E2A-PBX, as revealed by ChIP. Shown are mean and SD (n=3).

B. Significant reduction of MLL binding to *Hoxa9* and *Meis1* loci was detected in MLL-AF9-ER transformed cells 4 days after tamoxifen (4OHT) withdrawal, as revealed by ChIP using anti-MLL antibody. Shown are mean and SD (n=3).

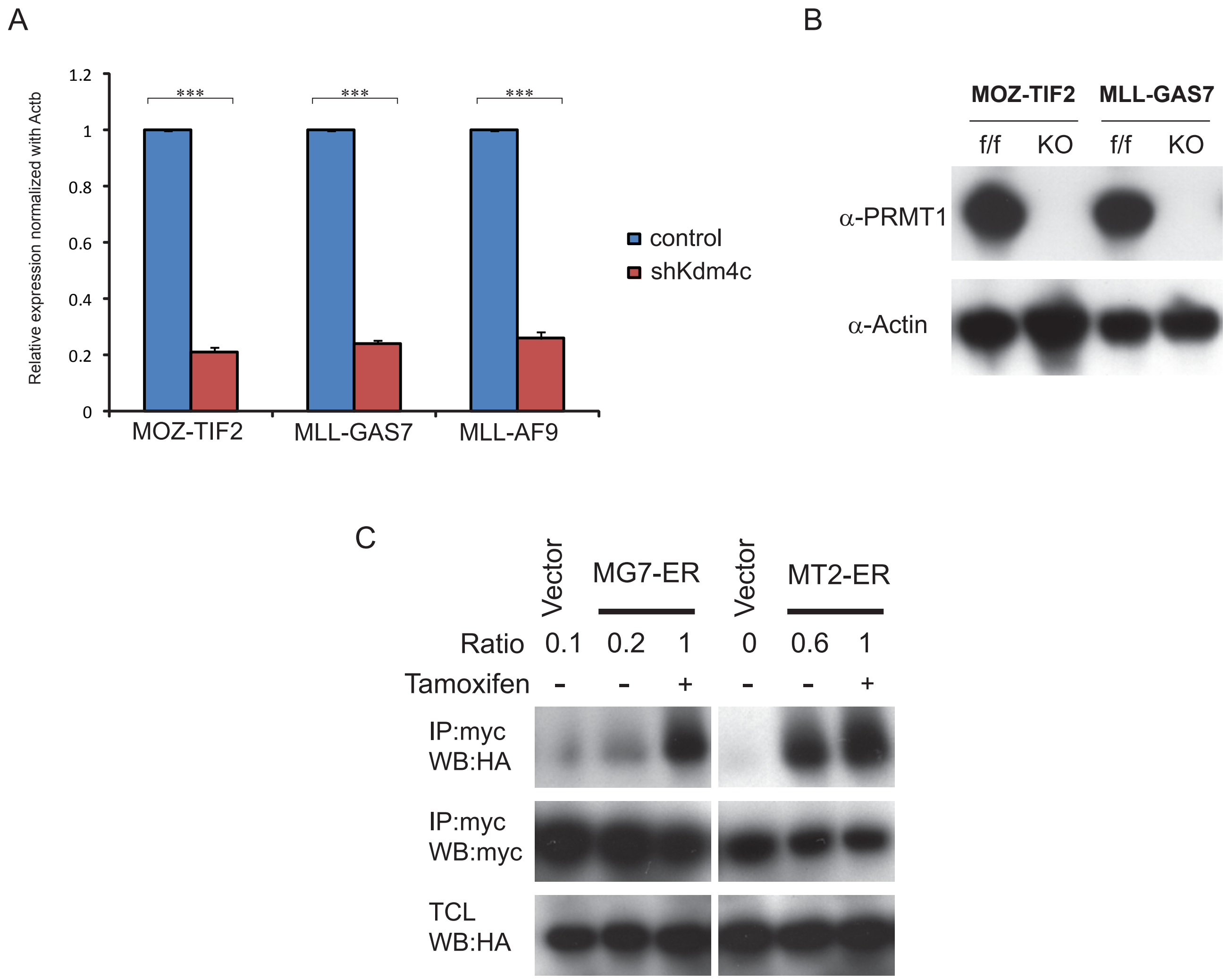

Figure S4, related to Figure 4. Validation of Kdm4c knockdown and Prmt1 knock-out in RNA-seq samples

A. RT-qPCR validation of Kdm4c knockdown (shKdm4c) on MOZ-TIF2, MLL-GAS7 and MLL-AF9 leukemic cells prior to RNA-Seq analysis. Shown are mean and SD (n=3).

B. Western blot shows the loss of Prmt1 protein expression in MOZ-TIF2 and MLL-GAS7 leukemic cells after knockout (KO), compared with floxed control (f/f).

C. FLAG tagged inducible MLL-GAS7-ER or MOZ-TIF2-ER were co-transfected with myc-KDM4C and HA-Prmt1 in HEK293 cells followed by co-immunoprecipitation analysis. Activation of MLL-GAS7 and MOZ-TIF2 leukemic fusions by tamoxifen resulted in increasing co-recruitment of Prmt1 by Kdm4c comparing with inactive complexes. The band intensity was normalized to control cells expressing active leukemic fusions.

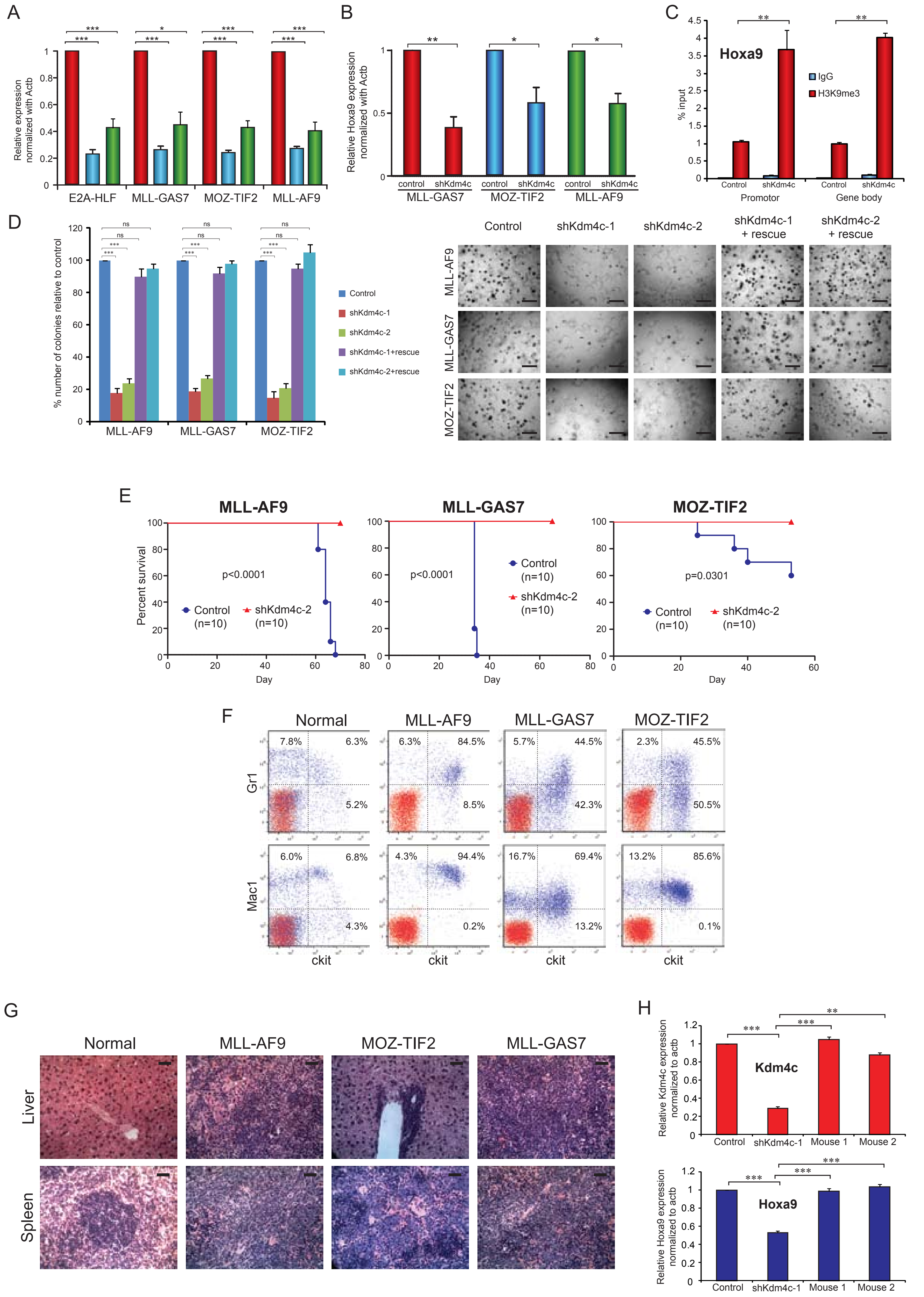
A
Relative expression normalized with Actb
E2A-HLF
MLL-GAS7
MOZ-TIF2
MLL-AF9
B
Relative Hoxa9 expression normalized with Actb
control
shKdm4c
C
Hoxa9
% input
IgG
H3K9me3
Promotor
Gene body
D
% number of colonies relative to control
Control
shKdm4c-1
shKdm4c-2
shKdm4c-1+rescue
shKdm4c-2+rescue
shKdm4c-1 + rescue
shKdm4c-2 + rescue
E
Percent survival
Day
p<0.0001
p=0.0301
Control (n=10)
shKdm4c-2 (n=10)
F
Normal
Gr1
Mac1
ckit
G
Liver
Spleen
H
Relative Kdm4c expression normalized to actb
Kdm4c
Relative Hoxa9 expression normalized to actb
Mouse 1
Mouse 2

Figure.S5

Figure S5, related to Figure 5. Effect of Kdm4c knockdown on leukemic transformation in vitro and leukemogenesis in vivo

A. Quantitative RT-PCR analysis showing the efficient knockdown of *Kdm4c* by two different shKdm4c shRNAs in different leukemia cells. Shown are mean and SD (n=3).

B. Quantitative RT-PCR analysis showing the significant reduction of *Hoxa9* expression in MLL-GAS7, MOZ-TIF2 and MLL-AF9 after Kdm4c knockdown by shKdm4c-1.

C. ChIP analysis showing the increased H3K9me3 mark at *Hoxa9* loci upon Kdm4c knock-down in MLL-AF9 leukemic cells.

D. Suppression of colony formation induced by MLL-AF9, MLL-GAS7 and MOZ-TIF2 is rescued by the co-expression of shRNA resistant human KDM4C. Shown are mean and SD (n=3). Scale bars indicate 50 μm.

E. Knockdown of Kdm4c in leukemic cells by shKdm4c#2 resulted in a significant increase in disease latency in MLL-AF9 (log-rank test p<0.0001), MLL-GAS7 (p<0.0001), MOZ-TIF2 (p=0.0301) mediated leukemia. Median disease latency: MLL-AF9: 64 days; MLL-GAS7: 34 days; MOZ-TIF2: 56 days; shKdm4c: undefined.

F. FACS analysis of the bone marrow of normal and leukemic mice as indicated showed the expression of c-kit and myeloid surface markers Gr1 and Mac1 in leukemia blasts.

G. Histological analysis revealed the infiltration of leukemic blasts into the liver and spleen of leukemic mice as indicated. Scale bar indicate 25 μm.

H. Quantitative RT-PCR validation of Kdm4c knockdown in leukemia cells isolated from disease mice. Analysis of the expression level of *Kdm4c* mRNA revealed the escape of Kdm4c knockdown (upper panel) and the upregulation of *Hoxa9* expression (lower panel) in the MLL-AF9 leukemic cells (Mouse 1 and 2).

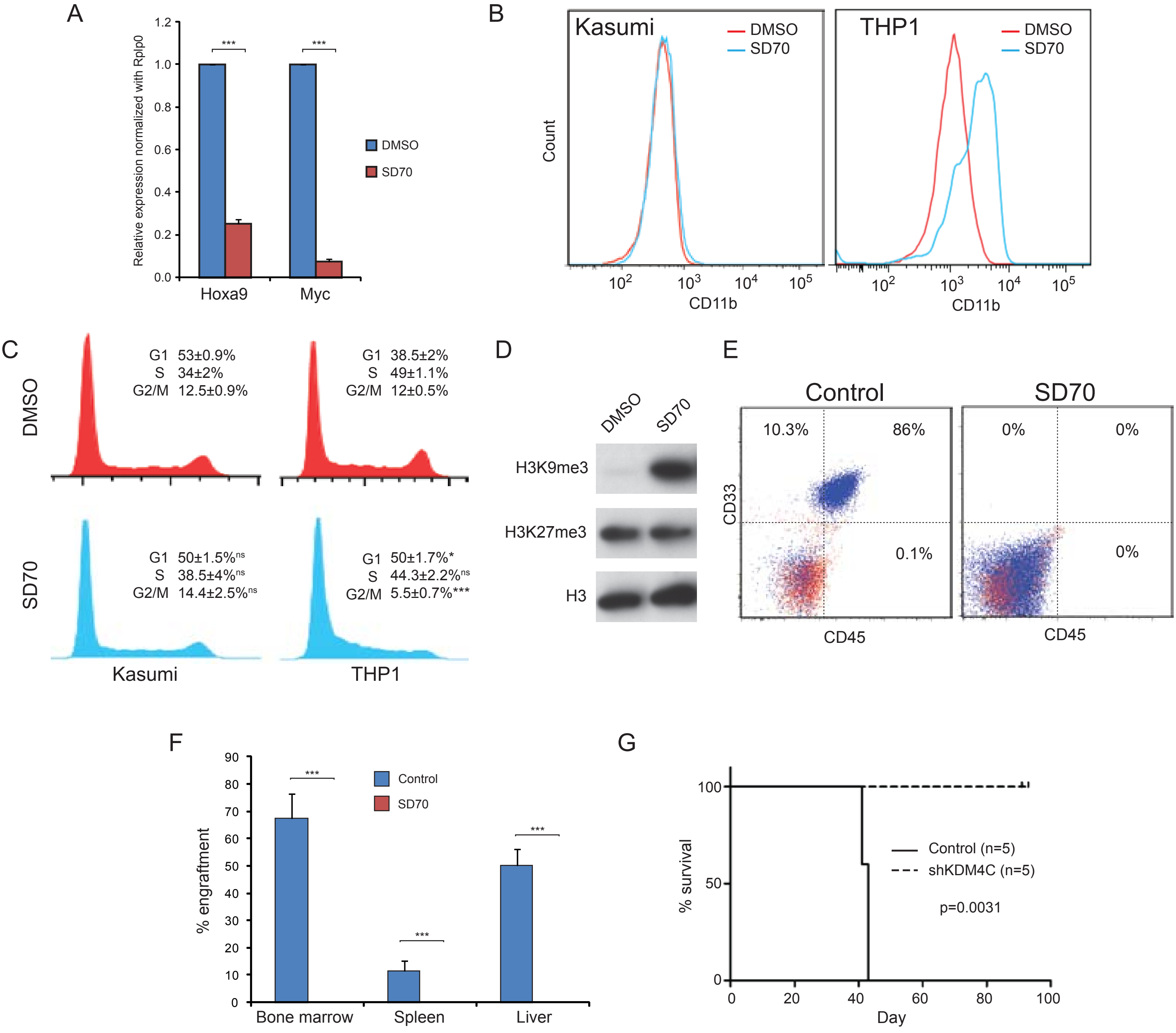

Figure S6, related to Figure 6. SD70 inhibits murine and human leukemogenesis.
A. Quantitative RT-PCR analysis showed that SD70 repressed the transcription activation of *Hoxa9* and *Myc* in murine MLL-AF9 leukemic cells after 3 days treatment. Shown are mean and SD (n=3).
B. Expression of myeloid surface marker CD11b was induced by SD70 after 3 days treatment in THP1 leukemia cells but not the control Kasumi cell lines, as revealed by FACS analysis.
C. Cell cycle analysis showing SD70 treatment as (B) induces an increase of G1 and reduction of S populations in THP1 carrying MLL-AF9 but not non-MLL rearranged Kasumi cells with AML1-ETO fusion.
D. Western blot of H3K9 and H3K27 methylation in SD70 treated human MLL leukemia cells for 3 days revealed the upregulation of H3K9me3 marks upon SD70 treatment as indicated in Figure 6H, which is consistent with the inhibition of KDM4C demethylation activity.
E. Representative FACS plot of bone marrow engraftment of human primary leukemia MLL3 in NSG mice from control and SD70 treated groups. They were analysed by FACS with human CD33 and CD45 staining. Engraftment of MLL3 leukemia cells was indicated by CD45+CD33+ population.
F. A summary of the percentage of MLL3 human primary leukemia engraftment in the bone marrow, spleen and liver in both the control and SD70 treatment cohort (n=5). SD70 treatment significantly inhibited the engraftment of human leukemia cells in the organs analysed (unpaired t-test; ***p<0.001).
G. Survival curves of in vivo xenograft study using MLL3 human primary leukemia cells transduced with scramble control or shKDM4C. NSG mice transplanted with MLL3 cells with scramble shRNA developed leukemia within 50 days, whereas cohort with KDM4C knockdown (shKDM4c) MLL3 cells had not succumbed to leukemia (log-rank test p=0.0031). Median disease latency: Control – 43 days; shKDM4C – undefined.

Supplemental Experimental Procedures

Plasmid constructs

Leukemia fusions and their deletion mutant constructs were cloned into MSCV retroviral vectors (Clontech) or pcDNA3-FLAG expression vector to generate FLAG-tagged expression plasmids. To generate myc-tagged expression constructs, genes of interest were subcloned into pCS2+MT vector. Most of the histone demethylase family genes were derived from IMAGE clones and subsequently subcloned into pRRL-3xFLAG lentiviral vector. Kdm4 demethylase family plasmids were kindly provided by Kristian Helin & Thomas Jenuwein; KDM3A/B were gifts of Zhang Yi. MSCV-MOZ-TIF2 is a kind gift from Brian Huntly. All plasmid constructs were confirmed by DNA sequencing. Prmt1 shRNA constructs were prepared as described (Cheung et al., 2007). Kdm4c shRNAs in pLKO lentiviral vector were kind gifts from Bill Hahn (shKdm4c TRCN0000103550, shKdm4c#2 TRCN0000103553 from Sigma Aldrich). Lentiviral vector harbouring luciferase reporter and hygromycin resistance gene (pCDH-CMV-luc-EF1-Hygro) was kindly provided by Lou Chesler.

Cell lines

Murine leukemia cell lines (E2A-PBX, MN1, MOZ-TIF2, MLL-AF9, MLL-ENL and MLL-GAS7) were established from retroviral transduction transformation assay (RTTA) as described (Zeisig and So, 2009) and cultured in RPMI with 20% FCS, 20% WEHI conditioned medium, 100 unit/ml penicillin and 100 μg/ml streptomycin (Sigma). Human leukemia cell lines NB4 was kindly provided by Arthur Zelent, THP1 by Mel Greaves, KASUMI by Olaf Heidenreich and K562, KG1, SEM, and HB11;19 were collected from ATCC. They were cultured in RPMI supplemented with 10% FCS, 100 unit/ml penicillin and 100 μg/ml streptomycin. Primary human patient samples (MLL1-3 and non-MLL1-3) were cultured in IMEM supplement with 10% FBS, 1X Glutamax (Invitrogen), 10ng/ml interleukin 3 (IL3), 10ng/ml interleukin 6 (IL6), 10ng/ml stem cell factor (SCF), 10ng/ml thrombopoietin (TPO), 10ng/ml FLT3 ligand (all human; Peprotech), 100 unit/ml penicillin and 100 μg/ml streptomycin.

Viral transduction and methylcellulose replating assay

RTTA was performed as previously described (Zeisig and So, 2009). Briefly, retroviral or lentiviral supernatants were collected 3 days after transfection of HEK293-GP cells (Clontech) or HEK293T respectively and used to transduce hematopoietic progenitors and stem cells positively selected for c-Kit expression by magnetic activated cell sorting (MACS; Miltenyi Biotec) from the bone marrow of 4-10 week old C57BL/6 mice or UbC-Luciferase transgenic mice kindly provided by Andrew Kung (Becker et al., 2006). After spinoculation by centrifugation at 500 × g for 2 hours at 32°C, transduced cells were cultured overnight in RPMI supplemented with 10% FCS, 20 ng/ml SCF, 10 ng/ml each of IL-3 and IL-6 (all murine; Peprotech). They were then plated in 1% methylcellulose (Stem Cell Technologies, Vancouver, BC, Canada) supplemented with the same cytokines plus 10 ng/ml GM-CSF (Peprotech) and 1 mg/ml geneticine or 30 μg/ml blasticidine (Life Technology) for positive selection of transduced cells. For co-transduction experiments of leukemia fusions with shRNA constructs, the cells were co-selected with 2 μg/ml puromycin and 1mg/mL geneticine. For Kdm4c knockdown rescue experiments, leukemia cells were cotransduced with Kdm4c shRNA and shRNA-resistant human KDM4C lentiviruses, and co-selected with 2 μg/ml puromycin and 30 μg/ml blasticidin. After 5-7 days culture colonies were counted to calculate the transduction efficiency. Single-cell suspensions (10^4 cells) of antibiotic resistant colonies were then replated in methylcellulose media supplemented with the same cytokines aforementioned without antibiotics. Subsequent replatings were usually repeated every 5-7 days.

In vivo Leukemogenesis assays

To generate full-blown murine leukemia cells ("leukemia cells" throughout the manuscript), 10^6 immortalized cells after the third round replating were injected via the tail vein into 6-10 weeks old syngeneic C57BL/6 mice which had received a sub-lethal dose of 5.25 Gy total body γ irradiation (^{137}Cs). To study the effect of down-regulation of Prmt1 and Kdm4c on leukemogenesis in vivo, the mice were injected with 10^5 murine full-blown leukemia cells as indicated. In vivo experiments with Prmt1 knockdown were transduced with either control vector or shPrmt1 retrovirus and sorted by eEGFP signal for transduced cells. Transduced Kdm4c knockdown cells (also control vector transduced) were antibiotic selected prior to transplantation. Prmt1 knockout achieved by tamoxifen

treatment were confirmed by PCR genotyping prior to transplantation. Mice were maintained and monitored for development of leukemia by FACS analysis. Tissues were fixed in buffered formalin, sectioned and stained with hematoxylin and eosin for histological analysis. For primary human sample MLL3, cells were transduced with control plasmid or shKDM4c plasmid. Transduced cells were antibiotic selected for 3 days and 10^5 selected cells were transplanted by intra-femoral injection into sublethally irradiated immunodeficient *NOD/SCID/IL2Rg-/-* (NSG) mice. For in vivo experiments involved bioluminescence imaging and quantification of the leukemia burden, murine leukemias were transformed in c-Kit positive bone marrow cells from Ubc-luciferase reportor C56BL6 mice (Becker et al., 2006). Human leukemia was transduced with a lenitiviral luciferase reporter as indicated in the plasmid constructs section. Transplanted mice were injected with 150 mg/kg D-luciferin substrate intraperitoneally and bioluminescence image acquired using IVIS Lumina II® (Caliper; Perkin Elmer) with software Living Image® Verion 4.3.1. Briefly, D-luciferin was injected into the animals intraperitoneally 10 min before the imaging procedure. Animals were maintained in general anaesthesia by isoflurane and put into the IVIS chamber for photography and detection of photon emission (large binning, F=1.2, exposure time: 3 min). The leukemia burden were measured and quantified by the same software as instructed. All the animal works were performed according to the guidelines and regulations of Animal (Scientific Procedures) Act 1986.

GST-pulldown affinity assay

5 μg of GST fusion protein was incubated with 1 mg HeLa cell lysate in NP-40 lysis buffer (150 mM NaCl, 50 mM Tris (pH 8), 5 mM EDTA, Complete Protease Inhibitor (Roche), 1% NP-40) for 2 hr at 4°C, washed with lysis buffer, eluted with SDS sample buffer & finally resolved in 10% SDS-PAGE. Protein interaction was detected by Western blotting followed by ECL chemiluminescence kit (GE Healthcare Life Science) and developed on X-ray film.

Transfection & Immunoprecipitation

Subconfluent HEK293 cells were transfected using calcium precipitation and harvested after 36-48 hrs. For generic immunoprecipitation, transfected cells were lysed in NP-40 lysis buffer (150 mM NaCl, 50 mM Tris (pH 8), 5 mM EDTA, Complete Protease Inhibitor (Roche), 0.1% -0.5% NP-40) for 30 min at 4°C, the lysates were cleared by centrifugation at 4°C. Cell lysates were incubated with the respective antibody overnight and then precipitated with protein-A/G Dynabeads (Life Technologies) at 4°C for 4 hr. For immunoprecipitation of FLAG-tagged protein, ANTI-FLAG M2 affinity agarose gel was used (Sigma Aldrich). Eluted proteins were resolved by SDS-PAGE and proteins of interest were detected with the corresponding antibody followed by Western blotting. Antibodies used for Western blotting are shown in Table 1. Densitometry analysis on immunoblot was performed with ImageJ software (Ver 1.49) according to their instructions.

Quantitative real time RT-PCR

Total RNAs extraction was performed using RNeasy kit (Qiagen) and treated with DNase (Ambion) or Nucleospin RNA extraction kit (Macherey-Nagel). cDNA was prepared using SuperscriptIII reverse transcriptase with random hexamer (Life Technology). Quantitative real time PCR was prepared with either FAST SYBR-green or Taqman probe based chemistry (Applied Biosystems) with StepOnePlus Real-Time PCR system (Applied Biosystems). RT-qPCR experiments were run in triplicate using duplicated experimental samples and analysed by ΔΔCT method. Primer sequences are shown in Table 2.

Generation of conditional Prmt1 knockout mice

Conditional *Prmt1* knockout mouse models have been generated using targeted ES cell clones of C57BL/6 background provided by European Conditional Mouse Mutagenesis program (EUCOMM). Microinjections of the ES clones and the PCR confirmation of germline transmission were carried out by the Mammalian Genetics Unit in the Medical Research Council. The *Prmt1* allele is specifically targeted with two *loxP* sites flanking exon 5 and 6, which encode part of the methyltransferase domain. Homozygous $Prmt1^{flox/flox}$ mice were mated with heterozygous Rosa26-Cre-ER mice (Jackson Lab) to generate the conditional $\text{Prmt1}^{\text{flox/flox}}$ Cre-ER/+ mice. All genotypes were confirmed by PCR. Deletion of *Prmt1* in the established leukemia cell lines was induced by tamoxifen at a concentration of 50ng/ml (Sigma) after 72-96 hours.

Chromatin Immunoprecipitation

Chromatin immunoprecipitation were performed as described (Cheung et al., 2007). Briefly, cells were fixed with 1% formaldedyde for 10-20 min at room temperature and then quenched with 0.125 M glycine for 5 min. To generate DNA fragments of 0.2-1 kb, 1-10x10^6 cells were sonicated by Bioruptor Nano (Diagenode) on maximum power for 10 min with 30s on-off interval. Chromatin fragments were incubated with the antibody overnight and collected in protein-A/G dynabeads (Life Technologies). Cross-linked products were reversed by heating overnight at 65°C, incubated with RNase for 1 hr at 37°C and then treated with proteinase K at 45°C for 1 hr. Eluted DNA was purified using QIAquick PCR purification kit (Qiagen) and used for quantitative PCRs with SYBR green or Taqman according to the manufacturer's protocol. For the inducible MLL-GAS7-ER and MOZ-TIF2-ER co-immunoprecipitation experiment, 50 ng/ml tamoxifen was added to the culture medium for activation of the leukemia fusions. Antibodies used for ChIP experiment are shown in Table 1. Primer sequences are shown in Table 3.

Flow cytometry analysis and sorting

Flow cytometry analysis of murine leukemia were performed as described (Yeung and So, 2009) using c-Kit (2B8), Gr-1 (RB6-8C5), Mac-1 (M1/70), CD4 (GK1.5), CD8(53-6.7), B220 (RA3-6B2), CD45.1 (A20) and CD45.2 (104) antibodies (BioLegend) and analysed by BD LSRII (Cheung et al 2007). FACS staining of human leukemia cells were performed using CD11B (ICRF44), CD14 (HCD14), CD33 (WM53), CD34 (581), CD38 (HB-7) and CD45 (H130) antibodies (BioLegend). To investigate the leukemic mice after transplantation, cells in bone marrow, spleen and liver were analysed. The donor cells were detected by $CD45.1^+CD45.2^-$ population for murine leukemia, or CD45+CD33+ population for human leukemia. GFP-positive cells were sorted using BD FACSAria cell sorter. AnnexinV staining is performed according to manufacturer protocol (eBioscience). For cell cycle analysis, cells were fixed in cold 70% ethanol overnight, washed with PBS and treated with 100ug/ml RNAse plus propidium iodide at 37°C for 30 min. The cell cycle profiles were analysed using FlowJo software (Ver 7.6.5).

Analysis of normal hematopoiesis in vitro and vivo

c-Kit enriched HSPC were isolated from the bone marrow of SJL mice (CD45.1) and transduced with control or shKdm4c lentivirus. After 48 hours puromycin selection, 1000 transduced cells were plated into methylcellulose containing GM-CSF, SCF, IL3 and IL6 for colony forming assays and CFU assays with additional 10ng/ml erythropoietin added. The number and types of colonies were counted after 1 week. To study the effect of *Kdm4c* knockdown on normal hematopoiesis in vivo, 2x10^5 transduced SJL donor cells (CD45.1) were transplanted together with 2x10^5 recipient bone marrow mononuclear cells into lethally irradiated C57BL/6 mice (CD45.2) by tail vein injection. Peripheral blood was collected after 6 weeks by tail vein bleeding and processed for the analysis of both myeloid and lymphoid population by FACS as described in the previous section (Yeung and So, 2009).

Nitro blue tetrazolium (NBT) reduction assay

NBT reduction assay was performed to determine myeloid differentiation. NBT was added to the liquid culture at a final concentration of 0.1% and incubated at 37°C CO_2 incubator for 3hrs. The differentiated cells were indicated by the deposition of dark blue insoluble formazan (NBT positive cells) and the percentage of differentiated cells were counted under microscopy. At least 200 cells were counted in most of the cases.

RNA sequencing and GSEA analysis

For RNA sequencing and GSEA analysis, MOZ-TIF2, MLL-GAS7 and MLL-AF9 leukemic cells were derived from c-Kit positive bone marrow cells from wild type (MLL-AF9) or the conditional $Prmt1^{flox/flox}$CreER (MOZ-TIF2 and MLL-GAS7) animals by RTTA. Leukemic cells were obtained from the bone marrow of recipient animals after primary transplantation. Suppression of *Kdm4c* in MOZ-TIF2, MLL-GAS7 and MLL-AF9 leukemic cells were achieved by shRNA knockdown. Inactivation of *Prmt1* was achieved by tamoxifen treatment for 3-4 days in vitro (MOZ-TIF2 and MLL-GAS7) and compared with corresponding leukemia cells treated by vehicle control.Total RNA was isolated using mirVana miRNA extraction kit (Ambion) or Nucleopsin RNA extraction kit (Macherey-Nagel). The RNA quality was determined by Bioanalyser 2100 (Agilent)

prior to library preparation. RNA library was prepared using Truseq Stranded RNA LT kit (Illumina) and next generation sequencing was performed with HiSeq 2000/2500 (Illumina). The quality control analysis was performed on all the RNA-Seq fastq files using FastQC software (Babraham Bioinformatics). The reads were aligned using TopHat2 (Kim et al., 2013) and counted with HTSeq (Anders et al., 2015). Further statistical comparisons were performed using DESeq or DESeq2 packages (Love et al., 2014). Comparative supervised heatmaps were generated using DESeq2 package. The desktop client of gene set enrichment analysis (GSEA) (Subramanian et al., 2005) was downloaded from Broad Institute website. The weighted GSEA analysis was performed on pre-ranked gene list with the reference c2.all.v4.0.symbols.gmt [Curated] gene list and 10000 permutations. The list of pathways was reported at FDR<0.05, complied and analysed by VennPlex (Cai et al., 2013) to generate Venn diagram comparing differential alteration in signalling pathways.

Table 1. List of antibodies used for Western blotting and ChIP analysis

Antibody	Cat. Number	Use	Company
FLAG (M2)	F1804	Western blotting	Sigma
Myc (9E10)	sc-40	Western blotting	Santa Cruz
Myc (A14)	sc-789	Western blotting	Santa Cruz
Actin	I-19	Western blotting	Santa Cruz
Sam68	sc-333	Western blotting	Santa Cruz
PSF	sc-28730	Western blotting	Santa Cruz
Prmt1	ab3768	ChIP	Abcam
Prmt1	07-404	Western blotting	Millipore
GST	sc-459	Western blotting	Santa Cruz
MLL	A300-086A	ChIP	Bethyl Laboratories
H4R3me2as	39705	ChIP & Western blotting	Active Motif
Kdm4c	A300-885A	ChIP	Bethyl Laboratories
Kdm4c	NBP1-49600	ChIP	Novis Biological
H3K9me3	ab8898	ChIP & Western blotting	Abcam
H3K9ac	ab4441	ChIP	Abcam
H3K27me3	07-449	ChIP	Millipore
H3	ab1791	ChIP & Western blotting	Abcam

Table 2. RT-qPCR primer sequences

qPCR primer	Forward	Reverse	Taqman probe (5' FAM, 3' TAMRA)
Hoxa9	CCGAACACCCCGACT TCA	TTCCACGAGGCACCAAA CA	TGCAGCTTCCAGTCCAAGGCGG
Meis1	CCTCGGTCAATGACG CTTTAA	TTTGAGAAATGTGAATTA GCTACTTGTACC	ACACCCCCTCTTCCCTCTCTTAGCA CTGA

Prmt1	TGTTTCACAATCGGCA TCTC	CCACTCGCTGATGATGAT GT	SYBR Green
Kdm4c	AGCATGGAAAGCGAC TTGAAA	TTGTGCCGGAGAAATGC AT	CCAAGGCTTCTTCCCCAGTAGCTC CC
Myc	AGCCCCTAGTGCTGC ATGA	GCCTCTTCTCCACAGACA CC	SYBR Green
Bcat1	GGGTTCCCTACTCCAC CTCT	CGGGGCTCAGGATCACA AAG	SYBR Green
Utx	GTCGAGCCAAGGAAA TTCA	GCAGGGATTACAGTCAA CCA	CGACTTGGGCTTATGTTCAAAGTG AACA
Actb	ACCTTCTACAATGAG CTGCGT	GCTGGGGTGTTGAAGGT CT	CCCTGAACCCTAAGGCCAACCGTG A
Gapdh	Applied biosystem	Cat. No 4351309	Taqman

Table 3. Primer sequences for ChIP analysis

ChIP Primer	Forward	Reverse
Hoxa9 promoter	GAATTTGCAGGGAAAGGAAA	GGCAGGAAGAAGAAAGTGGT
Hoxa9 gene body	TTCCCTCCATTTCTTGCTTT	GCCTTTGATCACATCTCCAC
Meis1 promoter	ACTGGCTGGTTGGAGACTTT	AGCTCCCAGTTCCAGAGAAA
Meis1 gene body	AATGGGCAGGAGTTAAGGAG	TCCACGCACTGTGAATTGTA
Gapdh	AGCTGTAAGCCATGCTGTGT	GTTGTCATGGCAGCAGAAAC
Rhdopsin	CACAGGGTACTGGCTTCTTG	CGTGTGTGAAACATCCACTG
Myc promoter	CCACAGGGGCAAAGAGGATT	AGGAGTCTCTGCCGGTCTAC
Myc gene body	GGATTTCCTTTGGGCGTTGG	CTAACCGGCCGCTACATTCA

References

Anders, S., Pyl, P. T., and Huber, W. (2015). HTSeq-a Python framework to work with high-throughput sequencing data. Bioinformatics *31*, 166-169.

Becker, C. M., Wright, R. D., Satchi-Fainaro, R., Funakoshi, T., Folkman, J., Kung, A. L., and D'Amato, R. J. (2006). A novel noninvasive model of endometriosis for monitoring the efficacy of antiangiogenic therapy. Am J Pathol *168*, 2074-2084.

Cai, H., Chen, H., Yi, T., Daimon, C. M., Boyle, J. P., Peers, C., Maudsley, S., and Martin, B. (2013). VennPlex--a novel Venn diagram program for comparing and visualizing datasets with differentially regulated datapoints. PloS one *8*, e53388.

Cheung, N., Chan, L. C., Thompson, A., Cleary, M. L., and So, C. W. (2007). Protein arginine-methyltransferase-dependent oncogenesis. Nat Cell Biol *9*, 1208-1215.

Kim, D., Pertea, G., Trapnell, C., Pimentel, H., Kelley, R., and Salzberg, S. L. (2013). TopHat2: accurate alignment of transcriptomes in the presence of insertions, deletions and gene fusions. Genome biology *14*, R36.

Love, M. I., Huber, W., and Anders, S. (2014). Moderated estimation of fold change and dispersion for RNA-seq data with DESeq2. Genome biology *15*, 550.

Subramanian, A., Tamayo, P., Mootha, V. K., Mukherjee, S., Ebert, B. L., Gillette, M. A., Paulovich, A., Pomeroy, S. L., Golub, T. R., Lander, E. S., and Mesirov, J. P. (2005). Gene set enrichment analysis: a knowledge-based approach for interpreting genome-wide expression profiles. Proceedings of the National Academy of Sciences of the United States of America *102*, 15545-15550.

Yeung, J., and So, C. W. (2009). Identification and Characterization of Hematopoietic Stem and Progenitor Cell Populations in Mouse Bone Marrow by Flow Cytometry. Methods Mol Biol *538*, 301-315.

Zeisig, B. B., and So, C. W. (2009). Retroviral/Lentiviral transduction and transformation assay. Methods Mol Biol *538*, 207-229.
